# Supplementary material for: Delivery of CAR-T cells in a transient injectable stimulatory hydrogel niche improves treatment of solid tumors
Source: Sci Adv. 2022 Apr 8;8(14):eabn8264. doi: 10.1126/sciadv.abn8264 (PMC8993118; doi:10.1126/sciadv.abn8264)
Supplement: 20220408-1 [file sciadv.abn8264.v1.pdf]

HEALTH AND MEDICINE

Copyright © 2022 The Authors, some rights reserved; exclusive licensee American Association for the Advancement of Science. No claim to original U.S. Government Works. Distributed under a Creative Commons Attribution License 4.0 (CC BY).

Delivery of CAR-T cells in a transient injectable stimulatory hydrogel niche improves treatment of solid tumors

Abigail K. Grosskopf[1]†, Louai Labanieh[2]†, Dorota D. Klysz[3], Gillie A. Roth[2], Peng Xu[3], Omokolade Adebowale[1], Emily C. Gale[4], Carolyn K. Jons[5], John H. Klich[2], Jerry Yan[2], Caitlin L. Maikawa[2], Santiago Correa[5], Ben S. Ou[2], Andrea I. d'Aquino[5], Jennifer R. Cochran[1,2], Ovijit Chaudhuri[6], Crystal L. Mackall[3,7,8,9]*, Eric A. Appel[2,5,7,8,10,11]*

Adoptive cell therapy (ACT) has proven to be highly effective in treating blood cancers, but traditional approaches to ACT are poorly effective in treating solid tumors observed clinically. Novel delivery methods for therapeutic cells have shown promise for treatment of solid tumors when compared with standard intravenous administration methods, but the few reported approaches leverage biomaterials that are complex to manufacture and have primarily demonstrated applicability following tumor resection or in immune-privileged tissues. Here, we engineer simple-to-implement injectable hydrogels for the controlled co-delivery of CAR-T cells and stimulatory cytokines that improve treatment of solid tumors. The unique architecture of this material simultaneously inhibits passive diffusion of entrapped cytokines and permits active motility of entrapped cells to enable long-term retention, viability, and activation of CAR-T cells. The generation of a transient inflammatory niche following administration affords sustained exposure of CAR-T cells, induces a tumor-reactive CAR-T phenotype, and improves efficacy of treatment.

INTRODUCTION

Adoptive cell therapy (ACT) is a promising new strategy to treat cancer that has been shown to be highly efficacious in the treatment of blood cancers. In the ACT process, immune cells are collected from a patient, isolated and engineered with receptors to recognize and eradicate cancerous cells, expanded to therapeutically relevant cell numbers, and then infused back into the patient for treatment. Chimeric antigen receptor (CAR)–T cells are engineered to target an antigen that is overexpressed on cancer cells. This strategy has been widely effective in treating various B cell malignancies, and several therapies have recently been approved by the U.S. Food and Drug Administration (*1*). Unfortunately, while numerous concerted efforts have sought to translate the successes of ACT into treatments for the multifarious solid tumors observed clinically (*2*), the traditional approaches to ACT have seen limited success in treating these solid tumors (*1*).

Currently, CAR-T cells are primarily delivered through intravenous infusion, which is effective in treating blood cancers because the T cells are able to readily find and eradicate cancerous cells. Unfortunately, large numbers of CAR-T cells are typically required for current treatment strategies to be successful, which requires costly, complex, and labor-intensive ex vivo expansion of the therapeutic cells that can inhibit their effector potential when transferred for treatment (*3*, *4*). Moreover, CAR-T cells administered in the typical way exhibit poor activity against solid tumors, as it is challenging for the cells to find, infiltrate, and expand within the typically immunosuppressive tumor microenvironment following systemic administration (*1*). In contrast, novel cell delivery methods, particularly those exploiting biomaterial scaffolds containing stimulatory molecules, can improve upon systemic delivery for treatment of solid tumors and elicit increased expansion of T cells at the injection site, improved tumor infiltration, and enhanced efficacy (*5*–*9*). Unfortunately, the biomaterial scaffolds developed for ACT to date often require complex manufacturing protocols that are not easily tuned to different solid tumor applications (*10*). Furthermore, these materials often require invasive surgical implantation procedures to reach tumor sites, hindering translation and limiting the use of these approaches to postresection applications, or have demonstrated applicability in immune-privileged tissues (*6*, *7*, *9*, *11*).

Effective therapy requires that CAR-T cells are highly activated at the tumor site (*12*, *13*), yet the high cytokine concentrations required for CAR-T activation lead to severe toxicities if delivered systemically, which typically precludes their use therapeutically. In current clinical approaches, T cells are expanded in high concentrations of cytokines and then isolated before administration into patients to avoid the toxicities associated with the cytokines. While locoregional delivery of cytokines can limit systemic cytokine exposure and reduce systemic toxicities (*14*), their low molecular weight and rapid elimination often require that the cytokines themselves be modified using protein engineering strategies to ensure sufficiently long-term local retention (*15*). The biomaterial scaffolds leveraged to date for locoregional delivery of CAR-T cells and cytokines have required the cytokines to be specially formulated either in microspheres or insoluble forms within the biomaterial to prevent rapid release (*7*, *9*).

On the basis of the promising results from previous studies of locoregional delivery of both CAR-T cells and cytokines (*7*–*9*), we

[1]Department of Chemical Engineering, Stanford University, Stanford, CA 94305, USA. [2]Department of Bioengineering, Stanford University, Stanford, CA 94305, USA. [3]Center for Cancer Cell Therapy, Stanford Cancer Institute, Stanford University School of Medicine, Stanford, CA 94305, USA. [4]Department of Biochemistry, Stanford University, Stanford, CA 94305, USA. [5]Department of Materials Science and Engineering, Stanford University, Stanford, CA 94305, USA. [6]Department of Mechanical Engineering, Stanford University, Stanford, CA 94305, USA. [7]Department of Pediatrics, Stanford University School of Medicine, Stanford, CA 94305, USA. [8]Stanford Cancer Institute, Stanford University School of Medicine, Stanford, CA 94305, USA. [9]Department of Medicine, Stanford University School of Medicine, Stanford, CA 94305, USA. [10]ChEM-H Institute, Stanford University, Stanford, CA 94305, USA. [11]Woods Institute for the Environment, Stanford University, Stanford, CA 94305, USA.
*Corresponding author. Email: eappel@stanford.edu (E.A.A.); cmackall@stanford.edu (C.L.M.)
†These authors contributed equally to this work.

hypothesized that next-generation biomaterials affording enhanced control over the co-delivery of CAR-T cells and immunostimulatory molecules will have the potential to realize effective ACT treatment strategies for solid tumors. For this approach, we drew inspiration from recent reports in the field of tissue engineering, which suggest that locoregional delivery of stem cells in physically cross-linked hydrogel scaffolds both improves cell viability during injection and enhances cell retention at the targeted injection site to facilitate local expansion of transplanted cells (*10*, *16*, *17*). In this work, we engineered a self-assembled and injectable biomaterials platform for CAR-T cell delivery based on Polymer-Nanoparticle (PNP) hydrogels (Fig. 1) (*17*–*22*). These hydrogels leverage highly scalable chemistries (*23*) and can be formulated under mild conditions that facilitate facile encapsulation of CAR-T cells and cytokines without the need for modification of the cargo. We show that the unique properties of these hydrogels enable facile administration via direct injection and the creation of a transient inflammatory niche in vivo that enhances CAR-T cell expansion and activation, profoundly improving efficacy in treating solid tumors in mice (Fig. 1A). We also demonstrate that similar efficacy is observed whether the treatment is administered proximal or distal to tumors, suggesting that this approach may be more broadly applicable for the treatment of metastases or tumors that are not easily accessible via direct injection or catheter delivery.

RESULTS

Development of an injectable hydrogel to act as an inflammatory niche

There are inherent materials design challenges in engineering hydrogels enabling slow delivery of diverse immunostimulatory molecules while maintaining CAR-T cell viability. We have recently shown that the dynamic polymer mesh within PNP hydrogels can be engineered to be small enough to provide prolonged retention of proteins while still enabling infiltration of immune cells (*24*). We hypothesized, therefore, that coencapsulation of immunostimulatory signals such as proliferative cytokines with CAR-T cells will enhance local T cell expansion following administration in the body.

To fabricate PNP hydrogels, a solution of dodecyl-modified hydroxypropyl methylcellulose (HPMC-C_{12}) can be simply mixed with a solution of biodegradable nanoparticles comprising poly(ethylene glycol)-*b*-poly(lactic acid) (PEG-PLA NPs) (figs. S1 and S2) (*17*, *18*). Upon mixing, dynamic multivalent and entropy-driven interactions between the HPMC-C_{12} polymers and the PEG-PLA NPs cause physical cross-linking and formation of a robust hydrogel material (Fig. 2). The self-assembled, entropy-driven cross-linking within these PNP hydrogels gives rise to temperature-invariant mechanical properties (*25*), and their physical cross-linking enables facile injection through a needle or catheter (*26*). The mesh size of PNP hydrogels can be engineered to be sufficiently small to retain local immunomodulatory signaling over prolonged and controlled time scales (*24*). To promote cellular motility and viability, the cell adhesion motif arginine-glycine-aspartic acid (RGD) can be attached to the hydrophilic corona of the PEG-PLA NPs using a "click" reaction before NP formation by nanoprecipitation of the PEG-PLA copolymers (fig. S1) (*17*, *27*). We have previously shown that cells can be easily suspended in RGD-PEG-PLA NP solutions before mixing with HPMC-C_{12} for facile encapsulation of viable therapeutic cells (*17*).

We first sought to determine how hydrogel formulation affects the mechanical properties of PNP hydrogels (Fig. 2 and fig. S3). In this work, we prepared three PNP hydrogel formulations referred to as PNP-1-1, PNP-1-5, and PNP-2-10, where the first number denotes

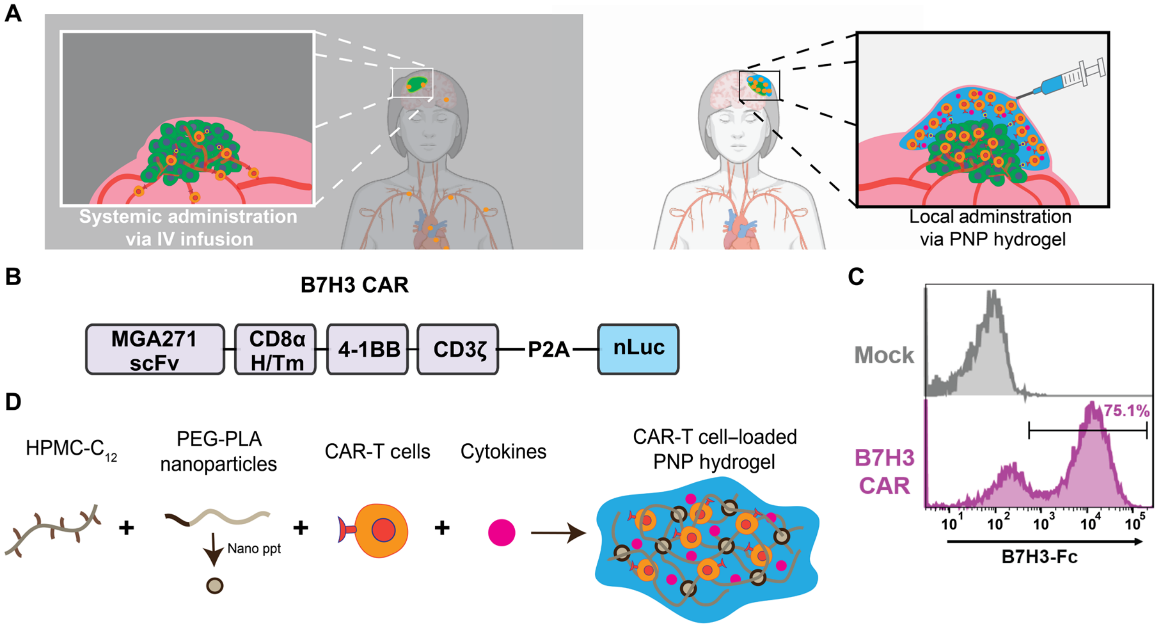

Fig. 1. Injectable hydrogels for creation of a local inflammatory niche for co-delivery for CAR-T cells and cytokines to improve treatment of solid tumors. (**A**) Schematic illustration demonstrating our proposed delivery method for CAR-T cells to solid tumors (right) compared to tradition intravenous (IV) approaches (left). (**B**) Schematic illustration of the B7H3 CAR construct used for all studies. (**C**) Transduction efficiency of B7H3 CAR-T cells as determined by staining with B7H3-Fc compared to nontransduced "Mock" T cells. (**D**) Formation of PNP hydrogels to coencapsulate CAR-T cells and stimulatory cytokines through self-assembly of dodecyl-modified hydroxypropyl methylcellulose (HPMC) and degradable block-copolymer nanoparticles.

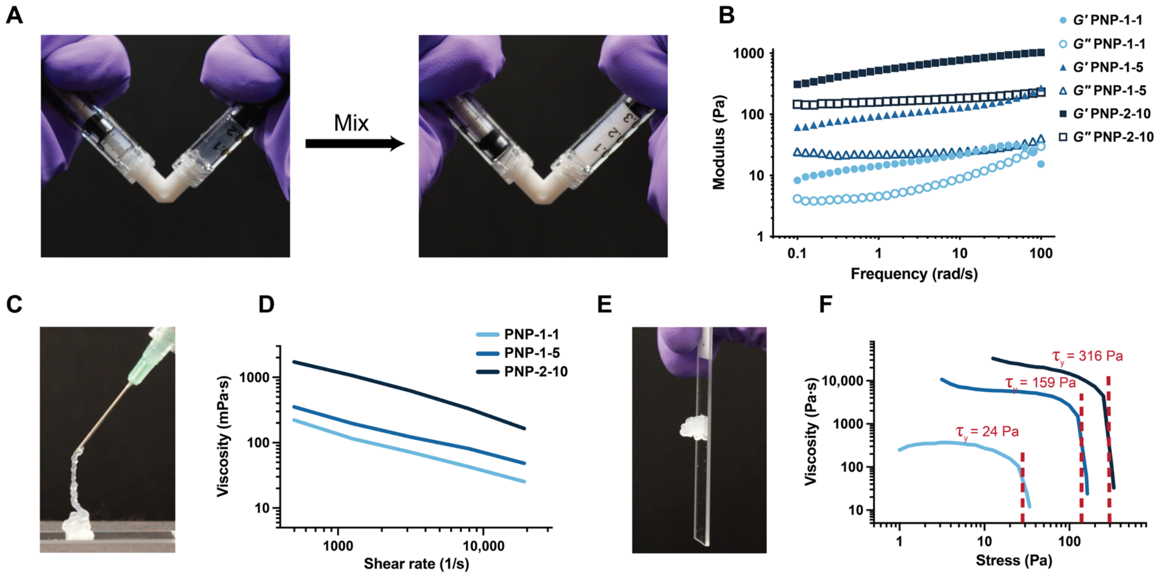

Fig. 2. Rheological properties of PNP hydrogels enable injectability and depot formation. (**A**) Facile formulation of PNP hydrogels by simple mixing of the biopolymer solution in one syringe (left) and the RGD-modified nanoparticles, cells, and cytokines in the other syringe (right) using a Luer lock mixer. After gentle mixing for 30 s, a solid-like PNP hydrogel encapsulating cells homogeneously is formed (right syringe). (**B**) Frequency sweep at 1% strain of three PNP hydrogel formulations, where the first number is the wt% HPMC-C_{12} polymer and the second number is the wt% NPs (remaining mass as saline). (**C**) Injection of cell-loaded PNP hydrogel through 26-gauge needle. (**D**) Flow sweep at high shear rates (representative of injection) for three PNP formulations. (**E**) A robust, solid-like hydrogel depot is formed that does not flow because of gravity. (**F**) Stress sweeps for three PNP formulations demonstrating tunable yield stress flow behavior. Yield stress transitions are noted with the red-dashed lines.

the weight % (wt%) of polymer and the second number denotes the wt% of nanoparticles (remaining mass is buffer). Oscillatory rheological testing demonstrated that these formulations all exhibit gel-like behavior across many time scales and up to high strains, as well as increasing stiffness with higher nanoparticle and polymer content (Fig. 2B, fig. S3). In addition, these formulations exhibited extreme shear thinning without fracture at high shear rates that are representative of the injection process (*26*). Note that the shear-thinning behavior of this class of hydrogel not only enables injection through small-diameter needles or catheters but also protects encapsulated cells from harsh mechanical forces during the injection process, leading to higher cell viability upon delivery (*28*, *29*). After injection, the formulations rapidly self-heal to form robust depots with substantial yield stress behavior that we hypothesized is critical for persisting in the subcutaneous space upon administration (Fig. 2F).

Prolonged retention of inflammatory cytokines in PNP hydrogels

It was first crucial that our materials platform exhibited degradation over relevant time scales for treatment of solid tumors. Previously described covalently cross-linked hydrogel systems do not decay over relevant time scales and may persist long past treatment, increasing patient burden (*7*, *9*). To evaluate the persistence of our physically cross-linked PNP hydrogel materials following implantation in immunocompetent mice, dye-labeled PNP-1-5 hydrogels were prepared by conjugation of a dye to the NPs used in fabrication of the materials and then implanted subcutaneously in mice by transcutaneous injection. The degradation of these materials was measured over time using an In Vivo Imaging System (IVIS; Fig. 3, A to C). PNP-1-5 hydrogels degraded within weeks of implantation and showed no visible signs of fibrotic capsule formation, exhibiting an average retention half-life of 8.9 ± 2.6 days, which is consistent with the typical time frame of a course of cancer treatment (*30*). These observations support previously published findings that PNP hydrogels do not elicit adverse immune responses or fibrotic capsule formation (*18*, *24*).

We hypothesized that coencapsulation of CAR-T cells and stimulatory cytokines such as interleukin-15 (IL-15), a potent T cell activator that has also been found to support maintenance of T cell memory (*31*), would improve CAR-T proliferation within the hydrogels. Previously developed biomaterial scaffolds have found improved T cell proliferation with use of IL-15 (*7*–*9*). Moreover, as IL-15 is a small protein (15 kDa) that exhibits a short elimination half-life of only 1.5 hours in humans, we hypothesized that it would benefit from sustained retention to improve its exposure profile (*31*). We therefore sought to determine whether PNP hydrogels could provide extended retention of IL-15 to improve CAR-T activation over longer time scales within the hydrogel following implantation. As mentioned above, cytokines can be easily encapsulated within these hydrogels by simple mixing during hydrogel fabrication without the need for complex formulation approaches or modification of the cytokine. We performed fluorescence recovery after photobleaching experiments to assess diffusion of fluorescein isothiocyanate (FITC)–labeled IL-15 and FITC-labeled dextran polymers close in molecular weight to IL-15 (e.g., 4 and 40 kDa). IL-15 exhibits a much lower diffusivity within PNP hydrogels than predicted for its low molecular weight and on the order of the self-diffusivity of the hydrogel mesh itself (Fig. 3D) (*21*), suggesting that the IL-15 is nonspecifically adhering to the hydrogel mesh (Fig. 3, D and E). This behavior likely arises from hydrophobic interactions between the IL-15 and the hydrophobically modified cellulosic polymer mesh of the PNP hydrogels (fig. S4A). In vitro release studies corroborated these

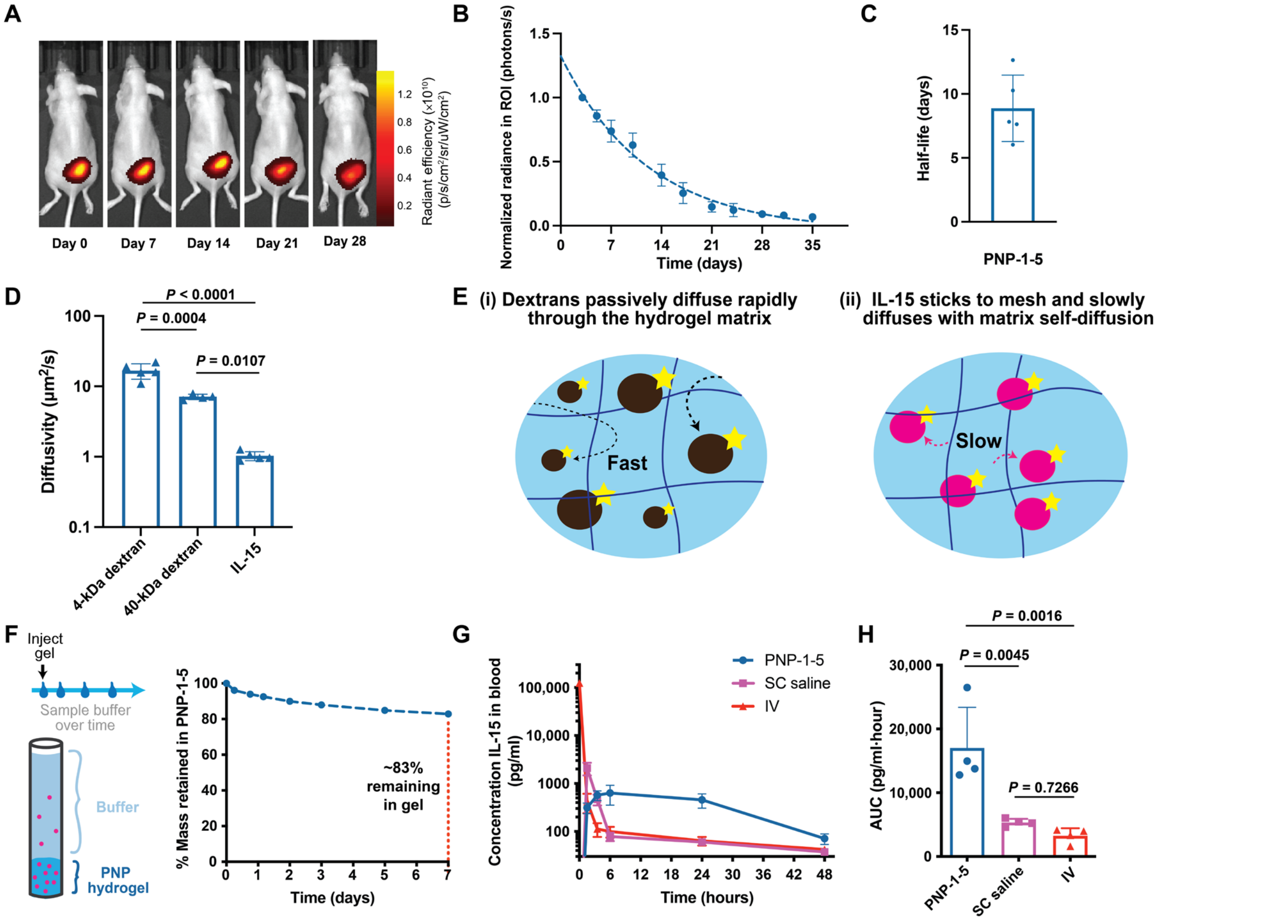

Fig. 3. PNP hydrogels enable prolonged retention of stimulatory cytokines and exhibit controlled degradation in vivo. (**A**) In vivo imaging of fluorescently tagged PNP-1-5 hydrogel over 4 weeks after subcutaneous injection in mice. (**B**) Quantification of fluorescence in the region of interest (ROI) surrounding the hydrogel and accompanying representative images over time. (**C**) Degradation half-life of PNP-1-5 hydrogels in vivo. (**D**) Diffusivity of FITC-dextran molecules and FITC-labeled IL-15 in the PNP-1-5 hydrogel formulation as measured by fluorescence recovery after photobleaching. (**E**) Schematic illustrating the different diffusion mechanisms of the (i) dextran molecules and (ii) IL-15 in the PNP hydrogel mesh. (**F**) Schematic of in vitro release assay of IL-15 from PNP-1-5 hydrogel immersed in saline over 1 week. %Mass of IL-15 remaining in the PNP-1-5 hydrogel during the release assay. (**G**) Pharmacokinetic curves of IL-15 in the blood administered intravenously, subcutaneously (SC), and from PNP-1-5 injected subcutaneously in mice. (**H**) Area under the curve (AUC) of the pharmacokinetic profiles of the various IL-15 administration routes.

observations, demonstrating that over 80% of the entrapped IL-15 was retained within the PNP-1-5 hydrogels after 1 week under infinite sink conditions (Fig. 3F). While the IL-15 may interact with the hydrogel components, it was found to be highly stable and remained active in PNP hydrogels over prolonged time frames under accelerated aging conditions (fig. S4B). Furthermore, we conducted a pharmacokinetic study of IL-15 in NOD scid gamma mouse (NSG) mice following standard administration of the cytokine in a saline bolus or encapsulated in PNP-1-5 hydrogels. Intravenous (IV) and subcutaneous (SC) saline injections of IL-15 resulted in high maximum serum concentrations (C_{max}), which is often associated with systemic toxicities, and rapid elimination. In contrast, sustained delivery of IL-15 from PNP-1-5 hydrogels significantly reduced the measured C_{max} values ($C_{max,PNP} = 570 \pm 130$ pg ml^{-1}, $C_{max,SC} = 2090 \pm 610$ pg ml^{-1}, $P = 0.0029$) and enhanced total IL-15 exposure ($AUC_{PNP} = 17{,}000 \pm 6400$ pg ml^{-1} hour^{-1}, $AUC_{SC} = 5330 \pm 580$ pg ml^{-1} hour^{-1}, $P = 0.0045$) (Fig. 3, G and H). Note that the release profile of IL-15 may be altered in immunocompetent mice because of the recruitment of endogenous immune cells to the inflammatory niche.

Development of an inflammatory niche for CAR-T cells

We next sought to determine how PNP hydrogel material properties affect CAR-T cell motility upon encapsulation (Fig. 4A). Increasing the polymer and NP content within these hydrogels reduces the dynamic mesh size of the matrix and increases hydrogel stiffness (*21*), both of which have been shown previously to affect the migration of T cells encapsulated within hydrogels (*27*). While PNP hydrogels exhibit a small (on the order of nanometers) yet dynamic hydrogel mesh that inhibits passive diffusion of protein cargo, we hypothesized that this mesh would still enable active migration of encapsulated T cells (*24*). To assess CAR-T cell motility within the PNP hydrogels, we conducted live cell motility experiments (Fig. 4, A to C). CAR-T cell migration speed decreased with decreasing hydrogel mesh size and increasing hydrogel stiffness ($F_{2,779} = 97.47$, $P < 0.001$), suggesting that migration and, therefore, retention within the hydrogel niche can be tuned through alteration of the hydrogel formulation (Fig. 4, B to E). We also found that RGD conjugation to the PEG-PLA NPs within the PNP hydrogel structure improves both T cell viability ($P = 0.0049$) and mobility ($P = 0.009$) (fig. S4, C to F).

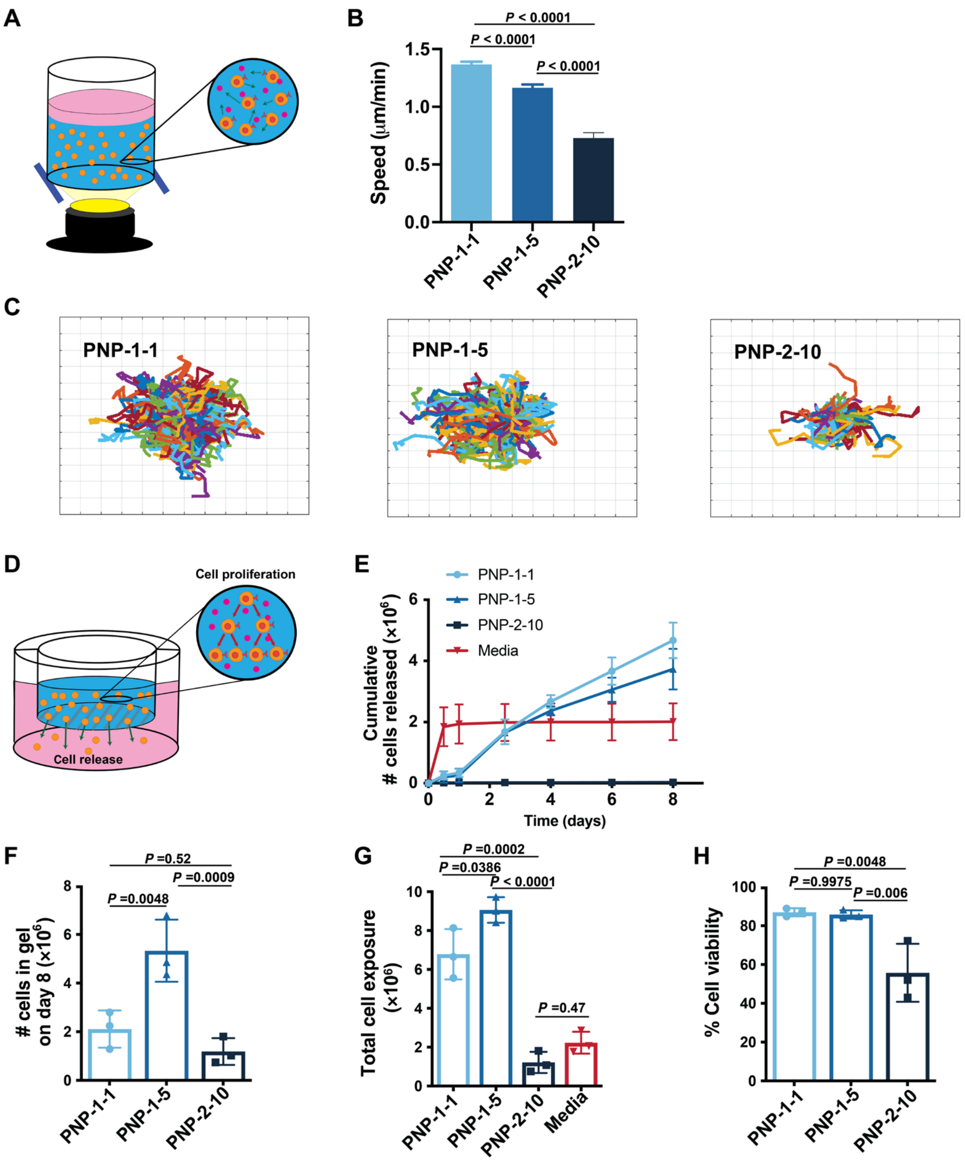

Fig. 4. PNP hydrogels control T cell motility and release. (**A**) Schematic illustration of in vitro experimental setup to evaluate CAR-T cell motility within PNP hydrogels. (**B**) CAR-T cell migration speeds in different PNP hydrogel formulations as quantified through cell migration experiments ($n > 150$ cells for all samples; means ± SEM). (**C**) Trajectories of migrating CAR-T cells within indicated formulations over 30 min. PNP-1-1, PNP-1-5, and PNP-2-10 hydrogel formulations were tested, where the first number is the wt% HPMC-C_{12} polymer and the second number is the wt% NPs. The trajectories are plotted at a common origin for easy visualization, where each grid is 50 μm wide. (**D**) Schematic of experimental setup to assess cell release from PNP hydrogel formulations. Hydrogel is injected into a porous transwell suspending over medium. Cells both proliferate in the hydrogel and escape into the medium below. (**E**) Cumulative number of cells released into the medium below over time of various PNP hydrogel formulations and a liquid medium control. At each time point, the medium below the transwell was replaced. (**F**) The total number of cells still remaining in the PNP hydrogel formulations at the end of the assay after 8 days, indicating whether proliferation within the hydrogel in addition to release has occurred. (**G**) The total cell exposure as quantified by the sum of the cumulative released cells and total cells in the transwell. (**H**) Cell viability of the cells remaining in the PNP hydrogel formulations in the transwell at the end of the cell release assay.

In contrast to tissue engineering applications in which cells are meant to remain within a scaffold, ACT requires that adoptive cells can effectively leave the depot. In addition to T cell motility within the hydrogels, we therefore also investigated migration of CAR-T cells out of the hydrogels. In these assays, CAR-T cells were loaded into various hydrogels and placed in transwells with a large pore size to allow T cells to escape the hydrogels and migrate into the medium below (Fig. 4D). The number of T cells that migrated into the medium over time was determined using cell counting assays. We found that the PNP-2-10 hydrogel did not allow cellular egress over an 8-day period, while the PNP-1-1 and PNP-1-5 continuously released T cells over time (Fig. 4E). In contrast, the media bolus control released all of the T cells into the medium within the first sampling period. At the end of the assay, when the hydrogels began to significantly degrade because of the immersion in media, hydrogels were dissociated, and encapsulated cells were examined. The PNP-1-5

hydrogel formulation promoted the formation of an inflammatory niche that effectively expanded viable (>85% viability) and proliferative CAR-T cells over the 8-day time frame while enabling controlled migration of cells out of the hydrogel (Fig. 4, F to H). In this way, the PNP-1-5 hydrogel enabled the highest total CAR-T cell exposure compared to the other hydrogel formulations and a 4.5-fold enhancement in total CAR-T cell exposure compared to standard bolus administration ($P < 0.0001$; Fig. 4, F to H). For these reasons, the PNP-1-5 formulation was chosen to evaluate further in efficacy studies in a murine cancer model.

Evaluation of locoregional depot-based CAR-T therapy for solid tumors

To assess the ability of PNP hydrogels to improve CAR-T cell treatment of solid tumors in vivo, human B7H3 CAR-T cells (2 million per dose) were encapsulated in PNP-1-5 hydrogels with and without coencapsulated IL-15 (0.25 μg per dose) and administered peritumorally in NSG mice bearing a subcutaneous human medulloblastoma (MED8A) solid tumor (Fig. 5A and figs. S5 and S6) (*32*, *33*). Hydrogel-based treatments were compared to relevant controls, including intravenous and peritumoral administration of CAR-T cells with and without IL-15 (0.25 μg per dose) in a saline vehicle. In these assays, both tumors and CAR-T cells were tracked and quantified using a dual luciferase in vivo imaging approach with IVIS. As this model often exhibits severe graft-versus-host disease (GVHD) on account of the adoptive transfer of human T cells to NSG mice (*34*), the length of the study is limited to 30 days.

While subcutaneous bolus and intravenous control treatments were only able to cure 10 and 40% of treated animals, PNP-1-5 hydrogel–based treatments cured 70% of treated animals ($P = 0.023$ and $P = 0.023$, respectively; table S1). Further statistical analysis including blocking for experimental variables (e.g., initial tumor sizes and experimental trials) concurred that delivery of CAR-T cells in the PNP-1-5 hydrogels resulted in a statistically significant faster time to cure compared to the subcutaneous saline or intravenous controls (fig. S7). Even fourfold higher CAR-T cell dosing administered subcutaneously or intravenously demonstrated the inability to cure and cured at a slower rate than PNP-1-5 hydrogel delivery, suggesting a dose-sparing effect where PNP-1-5 hydrogels carrying fewer cells outperformed these more traditional approaches to CAR-T administration (fig. S8).

Incorporation of IL-15 (0.25 μg per mouse) within the PNP-1-5 hydrogels alongside the CAR-T cells further improved treatment (Fig. 5, C and D). The dose of IL-15 used in these studies was below the maximum tolerated subcutaneous dose of recombinant human IL-15 rhIL-15 used in recent human clinical trials scaled to mice (Supplementary Discussion) (*14*, *35*, *36*), in contrast to many recent preclinical studies that use 5- to 20-fold higher concentrations of

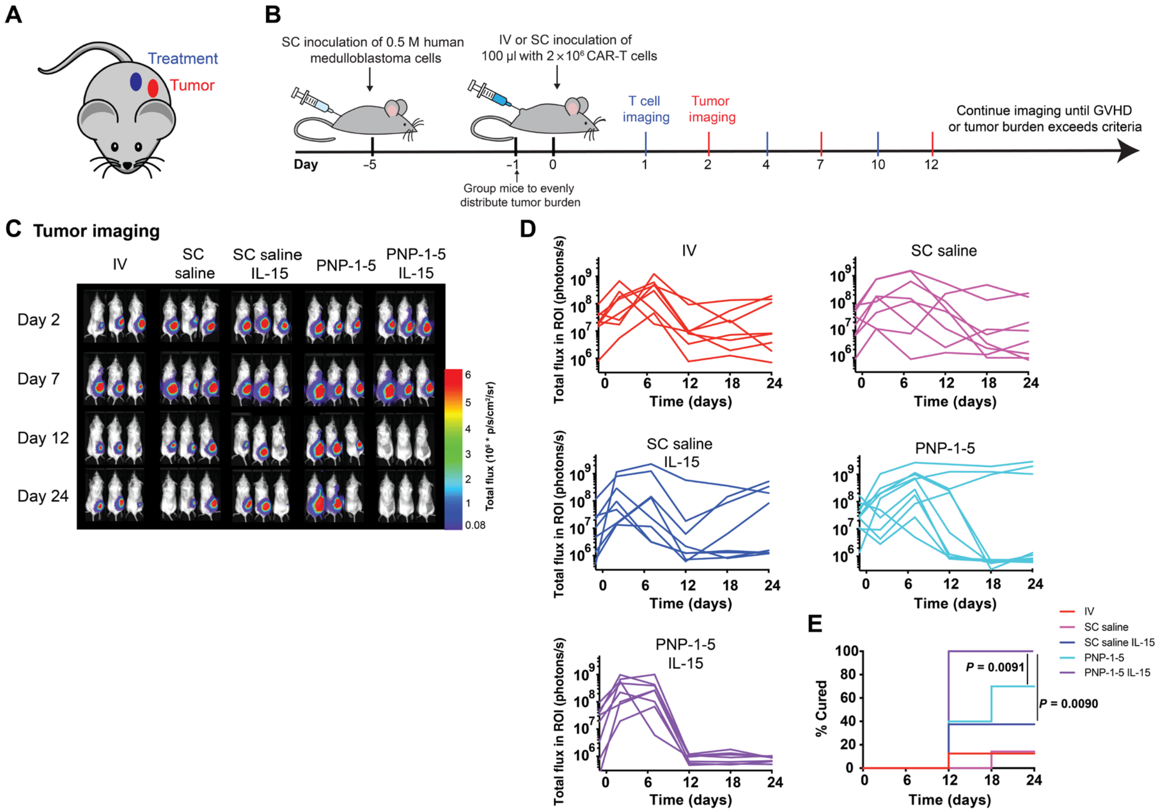

Fig. 5. PNP hydrogels improve treatment efficacy of solid tumors. (**A**) Schematic demonstrating placement of the tumor and the injected treatment. (**B**) Experimental timeline for cancer experiments treating subcutaneous human medulloblastoma in mice with 2×10^6 CAR-T cells administered with different delivery methods, including (i) intravenous bolus, (ii) subcutaneous bolus, (iii) subcutaneous bolus delivery containing 0.25 μg of IL-15, (iv) PNP-1-5 hydrogel, and (v) PNP-1-5 hydrogel containing 0.25 μg of IL-15. GVHD, graft versus host disease. (**C**) Luminescent imaging of tumors in all experimental groups at all time points. (**D**) Quantification of imaging data for all experimental groups ($n = 8$ to 10 for all groups over two experiments). (**E**) Percentage cured during the experiment across groups (defined as time when signal drops below and stays below a total flux of 2×10^6 photons/s). Further statistical analysis is reported in table S1.

IL-15 (*9*, *37*). Similarly, increased treatment efficacy was observed with co-delivery of IL-2 (0.25 μg per mouse) alongside CAR-T cells in PNP-1-5 hydrogels (fig. S9). Note that locoregional IL-15 delivery in PNP-1-5 hydrogels coupled with intravenous administration of "mock" CAR-T cells has no effect on tumor growth, confirming that CAR functionality is necessary for treatment and that the locoregional exposure of IL-15 at this dose is insufficient to produce antitumor responses. Overall, co-delivery of CAR-T cells with stimulatory cytokines in PNP-1-5 hydrogels improved the efficacy and consistency of treatment, with all mice being completely cured by day 12 (Fig. 5, C to E). While all mice treated with CAR-T cells and IL-15 in PNP-1-5 hydrogels were rapidly cured within the first 2 weeks, only 40% of mice receiving a subcutaneous bolus administration of cells and cytokines were cured by day 24 (P = 0.009; Fig. 5E). The efficacy of subcutaneous bolus administration of CAR-T cells was not significantly improved with co-delivery of IL-15 (P = 0.29), further highlighting the benefit of generation of a local inflammatory niche within our PNP-1-5 hydrogels, providing prolonged retention of IL-15 and CAR-T cells.

Enhanced T cell expansion was also observed when CAR-T cells were delivered in the PNP-1-5 hydrogels, particularly when co-delivered with IL-15 (Fig. 6, A and B). The PNP-1-5 formulation comprising IL-15 resulted in over a 100-fold increase in T cell expansion compared to the intravenous control (P = 0.0417). Imaging data demonstrated that, at initial time points, the primary location of T cell signal originates from the hydrogel depot on the right subcutaneous flank, but on days 15 to 21, the primary location of signal becomes the spleen (fig. S10), aligning with the time frame of tumor eradication. Histological analysis of the hydrogel depot confirmed that CAR-T cells are still present within the hydrogels several days after administration and demonstrated that no adverse immune response to the PNP hydrogel biomaterials were observable, consistent with previous findings (fig. S11) (*24*). Furthermore, analysis of inflammatory cytokines within the serum following treatment confirmed that locoregional hydrogel depot treatment did not elicit spikes in inflammatory cytokines that are often associated with systemic toxicities (fig. S12) despite the greatly enhanced antitumor responses that we observed. It is expected that the IL-15 would

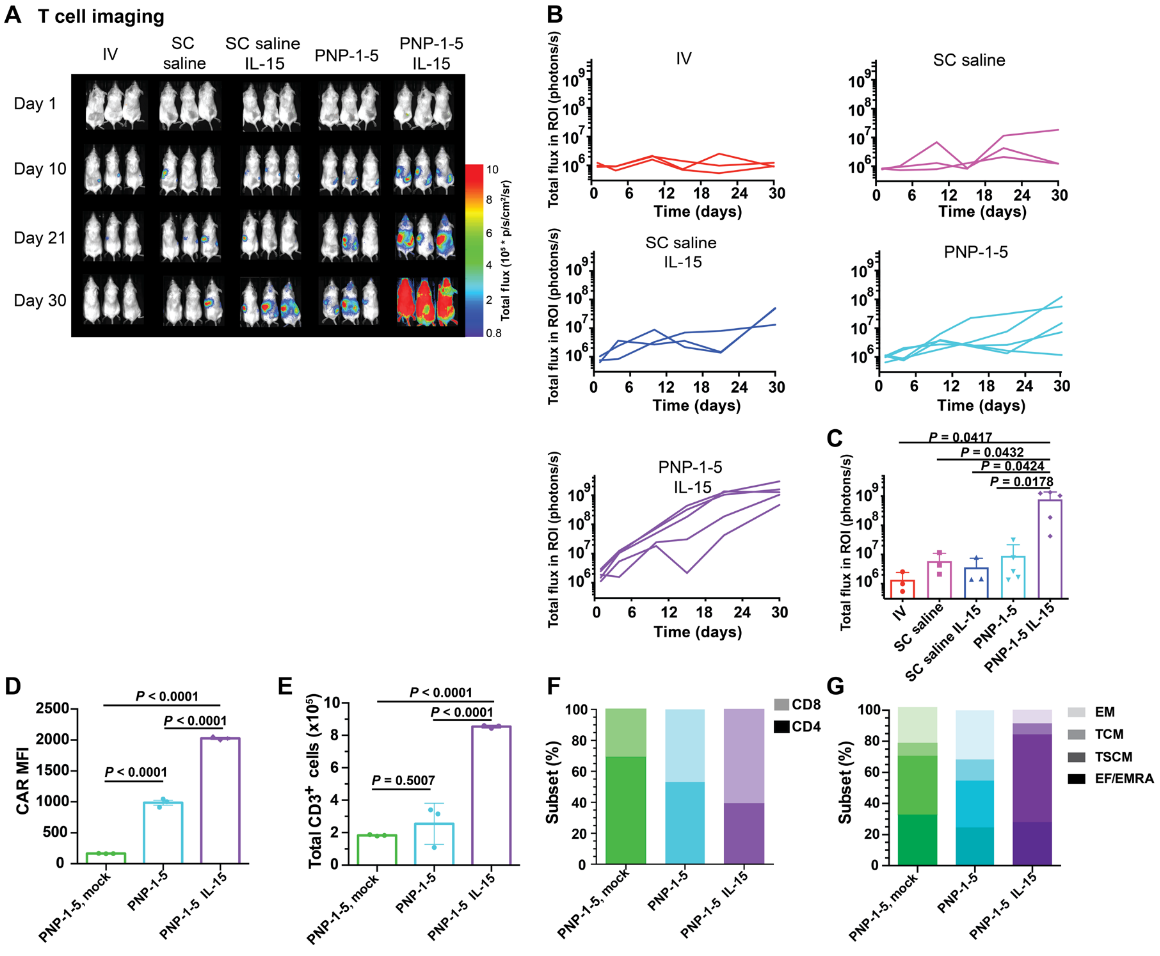

Fig. 6. PNP hydrogels improve expansion and phenotype of T cells. (**A**) Luminescent imaging of CAR-T cells in all corresponding experimental groups. (**B**) Quantification of imaging data for all experimental groups (n = 3 to 5 for all groups). (**C**) T cell signal across groups at day 21. Additional P values for comparisons between all treatment groups can be found in table S2. (**D**) CAR mean fluorescence intensity (MFI) (n = 3 for all groups; means ± SD), (**E**) total $CD3^+$ T cells (n = 3 replicates for all groups; means ± SD), (**F**) relative $CD4^+$ and $CD8^+$ content (mean of n = 3 for all groups with P < 0.0001 for all comparisons), and (**G**) memory CAR-T cell subsets (mean of n = 3 replicates for all groups with P values comparing groups shown in table S3) of ex vivo expanded CAR-T cells extracted from PNP-1-5 hydrogels 10 days after treatment in the MED8A tumor model. EM, effector memory; TCM, central memory; TSCM, T stem cell memory; EF/EMRA, terminally differentiated effector memory cells.

likely elicit responses in immunocompetent mice that are not observed in these immunocompromised NSG mice, as the stimulatory cytokine may elicit additional immune activation arising from endogenous immune cell recruitment to the gels, which may also affect the activation of the encapsulated CAR-T cells.

Evaluation of the impact of the local inflammatory niche on CAR-T phenotype

To better understand the mechanism for enhanced efficacy observed for co-delivery of CAR-T cells and IL-15 in a local inflammatory niche within PNP-1-5 gels, ex vivo analysis of T cells explanted from gels was performed 10 days after treatment. This analysis revealed enhanced CAR expression ($P < 0.0001$) (Fig. 6D), increased presence ($P < 0.0001$) (Fig. 6E), and greater proportions of $CD8^+$ ($P < 0.0001$) (Fig. 6F) and T stem cell memory subsets ($P < 0.0001$) (Fig. 6G) when CAR-T cells were coencapsulated with IL-15 compared to PNP-1-5 hydrogel alone (table S3), consistent with previously published findings (*38*, *39*). Nonsignificant changes to the proportions of central memory, effector memory, and terminally differentiated effector memory cell T cell subsets were observed when IL-15 was coencapsulated with CAR-T cells. Furthermore, while all CAR-T treatment groups evaluated expressed similar levels of the activation markers 4-1BB, LAG-3, and PD-1, co-delivery of IL-15 within the PNP-1-5 hydrogels resulted in increased expression of CD39 (fig. S13). This marker has recently emerged as an important marker of tumor-reactive T cells (*40*–*42*) but is also expressed on exhausted T cells (*42*). The regulation of CD39 expression has been shown to be dependent on hypoxia-inducible factor 1a activity (*43*, *44*), which can be induced by IL-15 signaling (*45*). Further studies are required to determine whether increased CD39 expression on CAR-T cells encapsulated in PNP-1-5 hydrogels with IL-15 is due to increased tumor reactivity, induction by IL-15 signaling, increased CAR-T exhaustion, or some other mechanism. All memory subsets were also found to be similar between all CAR-T groups evaluated in the spleen and blood (fig. S14).

Evaluation of distal depot-based CAR-T therapy for solid tumors

In addition to delivering hydrogel-based CAR-T treatments peritumorally, we also sought to evaluate whether the hydrogel-based inflammatory niche can exhibit potent antitumor efficacy when delivered subcutaneously on the distal flank of the mouse from the tumor (Fig. 7 and fig. S15). Distal administration ensures that CAR-T cells released from the hydrogel depot over time cannot drain directly to the tumor or the tumor-draining lymph node. We hypothesized that the enhanced expansion and maintenance of antitumor activation of the CAR-T cells enabled by the transient inflammatory niche within the hydrogels might elicit a potent abscopal effect relevant to metastatic cancer applications. In vivo imaging studies revealed that the hydrogel depot led to improved tumor clearance over a distal bolus control, although the two treatments exhibited similar overall T cell expansion and a similar distribution of T cell subsets in the blood and spleen (fig. S16). Mice in these studies were monitored over a longer duration than the studies described above, as they experienced significantly delayed GVHD. While all mice treated distally with CAR-T cells and IL-15 in PNP-1-5

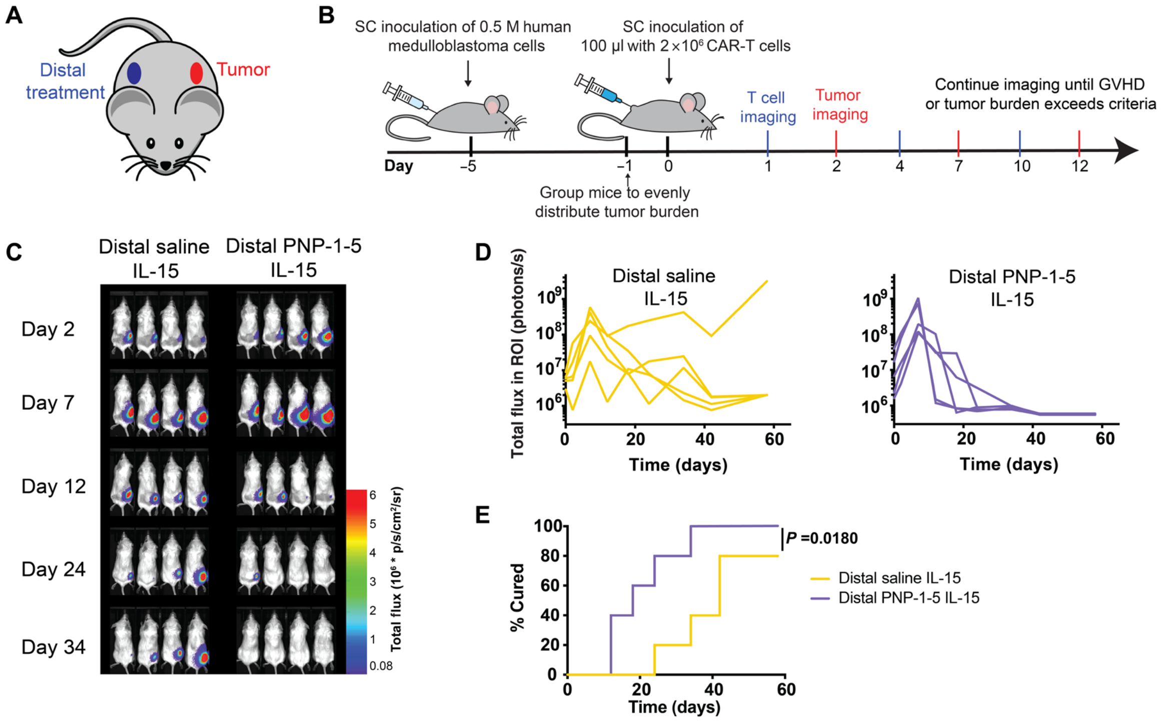

Fig. 7. PNP hydrogels are effective in treating distal subcutaneous human medulloblastoma in mice. (**A**) Schematic showing placement of subcutaneous tumor and distal subcutaneous treatments. (**B**) Schematic illustration of experimental timeline and treatment placement whereby mice received either (i) distal subcutaneous bolus injection of 2×10^6 CAR-T cells and 0.25 µg of IL-15 or (ii) PNP-1-5 hydrogel containing 2×10^6 CAR-T cells and 0.25 µg of IL-15. (**C**) Representative luminescent imaging of tumors with in vivo imaging ($n = 5$). (**D**) Quantification of imaging data for both experimental groups ($n = 5$). (**E**) Percentage cured during the experiment across groups (defined as time when signal drops below and stays below a total flux of 2×10^6 photons/s).

hydrogels were rapidly cured within the first month, only 80% of mice receiving a distal subcutaneous bolus administration of cells and cytokines were cured by day 60 ($P = 0.018$; Fig. 7E). Overall, these results are promising, as distal treatment with CAR-T cells and IL-15 within a transient inflammatory niche yields a potent abscopal effect that ultimately cures all treated animals, albeit slower than when this same treatment is given peritumorally. These results suggest that this hydrogel-based administration of CAR-T cells could have future use in treating metastatic cancers or inaccessible solid tumors.

DISCUSSION

While CAR-T therapy has resulted in remarkable antitumor effects in blood cancers, clinical successes in solid tumors have been severely limited, as these tumors often exhibit an unfavorable microenvironment that can inhibit T cell infiltration and/or promote T cell dysfunction (*1*). In this work, we describe a CAR-T delivery method leveraging an injectable PNP hydrogel depot technology to generate a local inflammatory niche to expand and maintain a reservoir of CAR-T cells and stimulatory cytokines to improve their antitumor efficacy. The facile assembly and administration of these materials using standard injection approaches shows great promise for locoregional delivery of the CAR-T therapy. Cell motility and cytokine release studies demonstrated that these PNP hydrogels afford a unique opportunity to simultaneously inhibit the passive diffusion of cytokines while permitting active motility of encapsulated CAR-T cells, enabling formation of a transient inflammatory niche upon injection that enhanced expansion of CAR-T cells in the body and induced a more tumor-reactive CAR-T phenotype. The in vivo expansion of more tumor-reactive CAR-T cells can potentially enable a crucial reduction in the number of therapeutic cells required for effective treatment, thereby reducing the high costs associated with the extended manufacturing periods currently required to generate sufficient cell numbers for standard treatment approaches. Furthermore, the success of distal treatment of tumors with this transient inflammatory niche holds promise for development of treatments for inaccessible or metastatic solid tumors.

This modular and versatile hydrogel depot technology is compatible with many cell and cytokine types, as encapsulation and sustained delivery does not require alteration of either the cells or the cytokines. This critical feature can potentially enable the use of these materials as the basis for treatments for many cancers. While PNP hydrogels have been developed previously for other applications, such as sustained delivery of vaccines, this work optimizes this unique self-assembled and injectable hydrogel platform for simultaneous delivery of therapeutic cells and immunostimulatory cytokines and to act as a local inflammatory niche to enhance the antitumor efficacy of transplanted cells. In future studies, we aim to investigate formulations comprising other immunostimulants and checkpoint inhibitors to modulate recruitment of endogenous immune cells to further amplify and expand antitumor responses. Moreover, additional studies are required to evaluate species-specific responses to the treatments that we describe here in fully immunocompetent mice (i.e., using mouse CAR-T cells to treat mouse tumors in immunocompetent mice). These studies will be required to elucidate both host effects on transplanted cells and niche effects on the host. Furthermore, additional studies evaluating approaches to maximize the potency of distal antitumor responses will be important for broadening the scope of these treatments, and we plan to assess this therapy's potential in many tumor types and in many parts of the body. Through using both human T cells and a human solid tumor model in this study, we hope that our findings constitute an important first step in evaluating the applicability of these niche-based treatments to the clinic (*1*). Overall, this interesting class of hydrogels addresses an unmet need for effective CAR-T cell delivery approaches that can enable the effective treatment of solid tumors.

MATERIALS AND METHODS

Materials

All chemicals, reagents, and solvents were purchased as reagent grade from Sigma-Aldrich, Acros, or Alfa Aesar and used as received unless otherwise specified. Glassware and stir bars were oven-dried at 180°C. When specified, solvents were degassed by three cycles of freeze, pump, and thaw.

HPMC-C_{12} synthesis

Hypromellose (HPMC; 1.5 g) was dissolved in *N*-methylpyrrolidone (NMP; 60 ml) by stirring at 80°C for 1 hour. Once the polymer had completely dissolved, the solution was cooled to room temperature. A solution of 1-dodecylisocyanate (0.75 mmol) dissolved in NMP (5 ml) was added to the reaction mixture followed by 150 μl of *N*,*N*-diisopropylethylamine as a catalyst. The solution was then stirred at room temperature for 16 hours. This solution was then precipitated in acetone, and the hydrophobically modified HPMC polymer was recovered by filtration, yielding HPMC-C_{12} (fig. S2). The polymer was then purified through dialysis [molecular weight cutoff (MWCO), 3000 g/mol] in Milli-Q water for 4 days and lyophilized to yield a white amorphous solid.

PEG-PLA synthesis

Following previously described protocols (*17*), PEG (0.25 g, 4.1 mmol) and 1,8-Diazabicyclo [5.4.0]undec-7-ene (DBU) [10.6 mg, 10 ml, 1.0 mole percent relative to Lactide (LA)] were dissolved in dichloromethane (DCM; 1.0 ml). LA (1.0 g, 6.9 mmol) was dissolved in DCM (3.5 ml) with mild heating. The LA solution was then added rapidly to the PEG/DBU solution and was allowed to stir rapidly for 10 min. The PEG-*b*-PLA copolymer was then recovered from the reaction medium by precipitation from excess 50:50 mixture cold diethyl ether and hexanes, collected by filtration, and dried under vacuum to yield a white amorphous polymer (M_n = 21 kDa, Đ = 1.08). Size exclusion chromatography traces were determined after passing through two size exclusion chromatography columns [resolve mixed bed low divinylbenzene, inner diameter of 7.8 mm, and weight-average molecular weight (M_w) range of 200 to 600,000 g mol^{-1} (Jordi Laboratories)] in a mobile phase of *N*,*N*-dimethylformamide with 0.1 M LiBr at 35°C and a flow rate of 1.0 ml min^{-1} (Dionex UltiMate 3000 pump, degasser, and autosampler; Thermo Fisher Scientific) (fig. S1).

RGD-PEG-PLA synthesis

Following previously described protocols (*17*), a 20-ml scintillation vial was charged with propargyl-functional cGGGRGDSP (22.7 mg, 26.6 μmol), azido-functional PEG-PLA (0.530 mg, 21.2 μmol), and NMP (4 ml). The reaction mixture was sparged with nitrogen for 10 min; then, a degassed solution (0.1 ml) containing CuBr (3.7 mg/ml) and tris-hydroxypropyltriazolylmethylamine (THPTA) (16 mg/ml) was added, and the reaction mixture was sparged with nitrogen for a further 10 min. The reaction mixture was incubated for 16 hours at room temperature; then, the polymer was precipitated from an excess of cold diethyl

ether in a 50-ml centrifuge tube and recovered by centrifugation. The polymer was then dissolved in acetone and precipitated again into diethyl ether, recovered by filtration, and dried in vacuum (fig. S1).

Nuclear magnetic resonance spectroscopy characterization

Deuterated solvents were purchased from Cambridge Isotope Laboratories and used as received. ^{1}H nuclear magnetic resonance (NMR) spectra were obtained and recorded on a Varian 600-MHz NMR spectrometer at 298 K, and chemical shifts (δ) are given in parts per million. ^{1}H NMR spectra were referenced to residual proton resonances in the deuterated solvents (figs. S1 and S2).

Nanoprecipitation

Procedure was followed and analyzed as described previously (*17*). A solution (1 ml) of PEG-PLA in acetonitrile (50 mg/ml) was added dropwise to water (10 ml) at a stir rate of 600 rpm. NPs were purified by ultracentrifugation over a filter (MWCO of 10 kDa; Millipore Amicon Ultra-15) followed by resuspension in water to a final concentration of 250 mg/ml. NP size and dispersity were characterized by dynamic light scattering (diameter, 35 nm; Polydispersity 0.02). A 50:50 mixture of RGD-functionalized PEG-PLA polymer (RGD-PEG-PLA) to unmodified PEG-PLA polymer was used to create the RGD-NPs.

Hydrogel formulation and cell encapsulation

Procedure was followed and analyzed as described previously (*17*). HPMC-C_{12} was dissolved in phosphate-buffered saline (PBS) at 6 wt% and loaded into a 1-ml Luer lock syringe. A cell pellet containing the number of cells to reach the desired concentration in the final hydrogels was suspended in PBS. A 20 wt% nanoparticle solution in PBS was then added to the cell suspension. A 30:70 mixture of RGD to plain NPs was used to form the hydrogel for studies containing RGD, yielding a 0.5 mM concentration of conjugated RGD in the gel. The CAR-T cell/nanoparticle solution was loaded into a 1-ml Luer lock syringe. The cell/nanoparticle syringe was then connected to a female-female mixing elbow, and the solution was moved into the elbow until it was visible through the other end of the elbow. The syringe containing the HPMC-C_{12} polymer was then attached to the other end of the elbow. The two solutions were then mixed gently back and forth through the elbow for 30 s to 1 min until the solutions had completely mixed and formed a homogeneous cell-loaded PNP hydrogel. IL-15 (R&D Systems) was incorporated with the nanoparticles during hydrogel formulation.

Rheological characterization of hydrogels

Rheological testing was performed using a 20-mm-diameter serrated parallel plate at a 600-μm gap on a stress-controlled TA Instruments DHR-2 rheometer. All experiments were performed at 25°C. Frequency sweeps were performed at a strain of 1%. Amplitude sweeps were performed at frequency of 10 rad/s. Flow sweeps were performed from low to high stress with steady-state sensing.

Viscometry at high shear rates

A RheoSense m-VROC viscometer was used to measure the hydrogel viscosity at high shear rates from low to high using a 1-ml Hamilton syringe. Each data point was collected at steady state.

Cell lines

MED8A was provided by S. Chesier (Stanford University, Stanford, CA). MED8A–green fluorescent protein (GFP)–Fluc cells were cultured in Dulbecco's modified Eagle's medium supplemented with 20% fetal bovine serum (FBS), penicillin (100 U/ml), streptomycin (100 μg/ml), 2 mM L-glutamine, and 10 mM Hepes (Gibco). Short tandem repeat (STR) DNA profiling was conducted once per year (Genetica Cell Line testing) and routinely tested for mycoplasma. Cell lines were cultured in a 5% CO_2 environment at 37°C.

Plasmid construction and virus production

B7H3 CAR–porcine teschovirus-1 2A (P2A)–Nluc plasmid was constructed by fusing the MGA271 scFv to CD8α hinge and transmembrane, 4-1BB costimulation domain, CD3ζ signaling domain, P2A ribosomal skipping sequence, and nanoluc in a mouse stem cell virus–based splice-gag vector (MSGV) retroviral vector (*46*–*48*). The Antares-P2A-mNG constructed was constructed by fusing a P2A sequence and mNeonGreen to the C terminus of Antares (*49*–*50*). Retroviral supernatant was produced using 293GP packaging cells transfected with the RD114 envelope plasmid and the corresponding plasmid construct, as previously described (*51*).

CAR-T cell isolation

T cells were isolated from buffy coats purchased from the Stanford Blood Center under an Institutional Review Board exempt protocol. Negative selection using a RosetteSep Human T cell Enrichment kit (Stem Cell Technologies) and SepMate-50 tubes was performed to purify primary human T cells. T cells were cryopreserved in CryoStor CS10 medium at a concentration of 1×10^7 to 2×10^7 cells/ml.

CAR-T manufacturing

Primary human T cells were thawed at day 0 and activated with anti-CD3/CD28 Dynabeads (Thermo Fisher Scientific) at a 3:1 bead–to–T cell ratio and cultured in AIM V + 5% heat-inactivated FBS, penicillin (100 U/ml), streptomycin (100 mg/ml), 2 mM L-glutamine, 10 mM Hepes, and rhIL-2 (100 U/ml). T cells were cultured in this medium containing IL-2 until day 10 and maintained at a concentration of 0.5×10^6 to 2×10^6 cells/ml. On day 2, virus-coated wells were prepared on 12-well non-tissue culture treated, RetroNectin-coated (Takara/Clontech) plates by spinning 1 ml of the corresponding virus on at 3200 rpm for 2 hours. T cells were then cultured on these plates for 24 hours. This transduction process was on day 3 after. Beads were magnetically removed on day 4, and cells were expanded until day 10 for in vivo experiments and days 10 to 14 for in vitro experiments. For dual virus cotransductions, T cells were transduced with Antares-P2A-mNG on day 2 and B7H3 CAR-P2A-Nluc on day 3.

Ex vivo CAR-T cell analysis

For ex vivo analysis of transferred T cells, mice were euthanized 10 days after T cell administration. Gels were harvested, and T cells were extracted by mechanical dissociation (gentleMACS dissociator, Miltenyi). Single-cell suspensions were filtered and stained for flow cytometry.

Flow cytometry

B7H3 CAR was detected using recombinant B7H3-Fc (R&D Systems) fluorescently labeled with the DyLight 650 Microscale Antibody Labeling Kit (Thermo Fisher Scientific). The following antibodies were used to stain T cells: BUV395 mouse anti-human CD4 (clone SK3, BD), BUV805 mouse anti-human CD8 (clone SK1, BD), BV605 mouse anti-human CD62L (clone DREG-56, BD), and BV711 mouse anti-human CD45RA (clone HI100, BD). CAR-T cell quantification

was performed using the CountBright Absolute Counting Beads (Thermo Fisher Scientific). Flow cytometry was performed on a BD LSRFortessa and analyzed on FlowJo version 10.7.1.

PNP hydrogel in vivo degradation study

Alexa Fluor 647 NPs were prepared using a combination of PEG-PLA (25 mg) and azide-PEG-PLA (25 mg). A 1-ml solution of combined PEG-PLA and azide-PEG–PLA in dimethyl sulfoxide (50 mg/ml) was added dropwise to 10 ml of water at room temperature under a high stir rate (600 rpm). NPs were purified by ultracentrifugation over a filter (MWCO of 10 kDa; Millipore Amicon Ultra-15) followed by resuspension in water to a final concentration of 200 mg/ml. The nanoparticles were then functionalized by mixing azide-functional NPs (250 μl, 20 wt%) with AF647-dibenzocyclooctyne (DBCO) (25 μl, 1 mg/ml) and waiting 12 hours. PNP hydrogels were made according to the procedures above using a PNP-1-5 formulation. Each 100-μl gel contained 5 μl of fluorescent AF647-conjugated NPs and 20 μl of nonfluorescently conjugated nanoparticles. Five SKH1-Elite Mice were each injected with 100 μl of fluorescently tagged PNP-1-5 hydrogel and imaged using the IVIS (Lago) over a series of time points spanning 35 days. When imaged, mice were anesthetized with isoflurane gas and imaged with an exposure time of 0.25 s, excitation wavelength of 600 nm, and emission wavelength of 670 nm (binning, medium; F/stop, 1.2). Total radiant efficiency [(photons/s)/(μW/cm^2)] was quantified using an equal-sized region of interest surrounding the gel depot. As early time points (time < 3 days) showed fluorescence in the region of interest to increase instead of decrease, fluorescent intensity at each time point was normalized to fluorescent intensity on day 3. Normalized fluorescence intensity values for each mouse (n = 5) between days 3 and 35 were fit to a single exponential decay model, and half-lives were acquired and averaged using GraphPad Prism.

CAR-T cell proliferation study

Promega CellTiter-Glo 3D Cell Viability Assay was used to characterize the short-term cell proliferation in different formulation conditions. Cells were encapsulated at 1 million cells per 100 μl in an opaque 96-well plate. Relative signal was measured after 2 days in culture by adding 100 μl per well of the CellTiter-Glo reagent, mixing for 5 min, allowing the plate to sit for 25 min, and then reading the luminescent signal with a 1-s integration time. Relative growth was calculated using a calibration curve generated by testing various known cell loadings in hydrogel.

CAR-T cell migration studies

Live cell imaging was done with a laser scanning confocal microscope (Leica SP8) fitted with temperature/incubator control, suitable for live imaging (37°C, 5% CO_2). A ×20 air objective, numerical aperture of 0.75, was used to acquire approximately 60-μm stack images for 5 hours (at 10-min intervals). Imaging parameters were adjusted to minimize photobleaching and avoid cell death. After completion of migration studies, the centroids of mCherry-labeled cells were tracked using the spots detection functionality in Imaris (Bitplane). Poorly segmented cells and cell debris were excluded from the analysis, and drift correction was implemented where appropriate. A custom MATLAB script was used to reconstruct cell migration trajectory.

CAR-T cell hydrogel release studies

CAR-T cells were loaded into PNP hydrogel formulations at 15 million cells/ml, and 100 μl of hydrogel was injected into transwell inserts. Transwells were gently placed into a 24-well plate, and 650 μl of medium was gently added below. The experiment was incubated according to standard culture conditions. At each time point, the medium was gently removed, the cells within the medium were counted, and the transwell was placed in a new well with new media. At the end of the assay (8 days, when hydrogel was beginning to noticeably dissociate), the contents of the transwell were diluted, cells were counted, and viability was recorded.

Fluorescence recovery after photobleaching

FITC-dextran (4 and 40 kDa, Sigma-Aldrich) was encapsulated at 1 mg/ml in PNP hydrogel formulations. IL-15 cytokine was labeled using a FITC Conjugation Kit (Fast)–Lightning-Link kit (Abcam) and encapsulated in PNP hydrogels at 8 μg/ml. Gels were placed onto glass slides and imaged using a confocal LSM 780 microscope. Samples were imaged using a low-intensity laser to observe an initial level of fluorescence. Then, the laser was switched to full intensity and focused on a region of interest with a diameter of 25 μm for 10 s to bleach a circular area. Fluorescence data were then recorded for 4 min to create an exponential fluorescence recovery curve. Samples were taken from different regions of each gel (n = 5 to 9). The diffusion coefficient was calculated according to

$$D = \gamma_D(\omega^2/4\tau_{1/2})$$

where the constant $\gamma_D = \tau_{1/2}/\tau_D$, with $\tau_{1/2}$ being the half-time of the recovery and τ_D being the characteristic diffusion time, both yielded by the ZEN software, and ω being the radius of the bleached region of interest (12.5 μm) (*24*, *52*).

In vivo experimental approaches

All animal studies were performed in accordance with the National Institutes of Health (NIH) guidelines, with the approval of the Stanford Administrative Panel on Laboratory Animal Care. MED8A-GFP-Fluc cells (5×10^5) were injected subcutaneously on the right flank of female NSG mice (the Jackson Laboratory, NSG 005557) 5 days before treatment in a 50:50 ratio of Matrigel (Cultrex PathClear) to PBS. One day before treatment, mice were imaged and distributed into groups of roughly equivalent tumor burden, assigned treatment randomly, and kept blinded until the end of the study. The minimum sample size per group for efficacy studies was determined using power calculations. CAR-T cells were encapsulated in PBS or PNP hydrogels in 1-ml syringes in a tissue culture hood 1 hour before injection as previously described. One syringe was prepared for each experimental group within a study. Extra gel (300 μl) was prepared in each syringe. Double the volume of bolus and intravenous controls was prepared. Syringes were transported on ice to the animal facility. PBS (100 μl) or PNP hydrogel was delivered to each mouse. Subcutaneous injections were delivered in a 21-gauge Luer lock syringe.

In vivo imaging

To image tumors, mice were intraperitoneally injected with D-luciferin, potassium salt (GoldBio) at 150 mg/kg in PBS. After 5 min, mice were anesthetized with isoflurane gas and imaged with an exposure time of 30 s with an IVIS (Spectral Imaging Instruments Lago-X). Signal was quantified as the total flux of photons/s in the region of interest at peak intensity. The region of interest was defined as a rectangular box of consistent size around the entire mouse. Background signal

for quantification was defined as the maximum signal observed through all imaging experiments in an equivalently sized rectangular box with no luminescent signal. To image tumors, mice were intraperitoneally injected with nano-luciferin (NanoLuc, Promega) at a 40× dilution in PBS. After 5 min, mice were anesthetized with isoflurane gas and imaged with an exposure time of 30 s with an IVIS (Spectral Imaging Instruments Lago-X). Signal was quantified as the total flux of photons/s in the region of interest at peak intensity. The region of interest was defined as a rectangular box of consistent size around the entire mouse. For analysis of specific regions (spleens and hydrogels), a consistent size box was placed in the region best estimated as the spleen and hydrogel for each mouse, and the resulting total flux in that region was recorded.

Histology

Gels were explanted through dissection from mice on day 5 of treatment and frozen in optimal cutting temperature compound. All samples were processed and stained by Stanford Animal Histology Services with hematoxylin and eosin staining and Cy5-CD3 staining. Two replicates were collected from PNP-1-5 hydrogel containing IL-15, and two replicates were collected for PNP-1-5 hydrogel. Representative images are shown.

Cytokine release in vitro

Capillary tubes were incubated with 0.1 wt% bovine serum albumin (BSA) overnight at 5°C. Capillary tubes were then dried using house air and evaporation and then loaded with 100 μl of PNP hydrogel containing 0.25 μg of IL-15. PBS (300 μl) was loaded on top of each gel. Samples were stored at 37°C to mimic physiological environments. At each time point, the PBS was completely removed using a long needle and stored at −80°C for later analysis. The PBS was then replaced. IL-15 concentrations were determined by enzyme-linked immunosorbent assay (ELISA) according to the manufacturer's instructions (R&D Systems Human IL-15 Quantikine Assay). Absorbance was measured at 450 nm in a Synergy H1 Microplate Reader (BioTek). At the end of 7 days, the gel was diluted by a factor of 10 in saline, allowed to incubate for 3 hours, and analyzed for the remaining cytokine. Cytokine concentrations were calculated from the standard curves. Mass in gel was calculated as the inverse of the total mass released into the release buffer during the study and the cytokine left in the gel at the end of the study.

Cytokine stability in vitro

IL-15 was encapsulated in PNP-1-5 hydrogel at 2.5 μg/ml. Hydrogel (100 μl) was loaded in low-binding Eppendorf tubes. Eppendorf tubes were incubated at 37°C and shaking at 150 rpm until various time points. At each time point, tubes were removed from the incubator, diluted by 10 with saline containing 0.1 wt% BSA and 5 wt% trehalose, and stored at −80°C until further analysis. IL-15 concentrations were later determined by ELISA according to the manufacturer's instructions (R&D Systems Human IL-15 Quantikine Assay). Absorbance was measured at 450 nm in a Synergy H1 Microplate Reader (BioTek). Cytokine concentrations were calculated from the standard curves.

Cytokine release in vivo

Serum was collected at the indicated times by tail vein blood collection and stored at −80°C. Serum IL-15 concentrations were determined by ELISA according to the manufacturer's instructions (R&D Systems Human IL-15 Quantikine Assay). Absorbance was measured at 450 nm in a Synergy H1 Microplate Reader (BioTek). Cytokine concentrations were calculated from the standard curves.

Inflammatory cytokine analysis

Blood (60 μl) was collected after 48 hours from each mouse (n = 3 for each group). Samples were processed and analyzed by the Stanford Human Immune Monitoring Center. Samples are reported relative to naive mice.

Statistical methods

All errors are reported as SDs unless reported otherwise. Sample size for each experiment is included in the corresponding section and the figure captions. Significance is reported with P values from a one-way analysis of variance (ANOVA) followed by Tukey's post hoc test for multiple comparisons. Statistical analyses on percentage cured plots were conducted using a log-rank Mantel-Cox test. To test whether cure time differed between treatments, we used a maximum likelihood parametric regression (SAS Release 3.8, Enterprise Edition) with censored data (PROC LIFEREG). Initial tumor size and experiment number were included in the model as fixed blocking effects to control for variation in initial tumor size or between experiments. Least-squares means were used to compare time to cure between individual treatments, and Tukey-Kramer post hoc tests were used to correct for multiple comparisons.

SUPPLEMENTARY MATERIALS

Supplementary material for this article is available at https://science.org/doi/10.1126/sciadv.abn8264

View/request a protocol for this paper from *Bio-protocol*.

REFERENCES AND NOTES

1. L. Labanieh, R. G. Majzner, C. L. Mackall, Programming CAR-T cells to kill cancer. *Nat. Biomed. Eng.* **2**, 377–391 (2018).
2. A. Titov, A. Petukhov, A. Staliarova, D. Motorin, E. Bulatov, O. Shuvalov, S. M. Soond, M. Piacentini, G. Melino, A. Zaritskey, N. A. Barlev, The biological basis and clinical symptoms of CAR-T therapy-associated toxicites. *Cell Death Dis.* **9**, 897 (2018).
3. A. Long, W. M. Haso, J. F. Shern, K. M. Wanhainen, M. Murgai, M. Ingaramo, J. P. Smith, A. J. Walker, M. E. Kohler, V. R. Venkateshwara, R. N. Kaplan, G. H. Patterson, T. J. Fry, R. J. Orentas, C. L. Mackall, 4-1BB costimulation ameliorates T cell exhaustion induced by tonic signaling of chimeric antigen receptors. *Nat. Med.* **21**, 581–590 (2015).
4. H. G. Caruso, R. Tanaka, J. Liang, X. Ling, A. Sabbagh, V. K. Henry, T. L. Collier, A. B. Heimberger, Shortened ex vivo manufacturing time of EGFRvIII-specific chimeric antigen receptor (CAR) T cells reduces immune exhaustion and enhances antiglioma therapeutic function. *J. Neurooncol.* **145**, 429–439 (2019).
5. P. S. Adusumilli, L. Cherkassky, J. Villena-Vargas, C. Colovos, E. Servais, J. Plotkin, D. R. Jones, M. Sadelain, Regional delivery of mesothelin-targeted CAR T cell therapy generates potent and long-lasting CD4-dependent tumor immunity. *Sci. Transl. Med.* **6**, 261ra151 (2014).
6. K. Wang, Y. Chen, S. Ahn, M. Zheng, E. Landoni, G. Dotti, B. Savoldo, Z. Han, GD2-specific CAR T cells encapsulated in an injectable hydrogel control retinoblastoma and preserve vision. *Nat. Cancer* **1**, 990–997 (2020).
7. S. B. Stephan, A. M. Taber, I. Jileaeva, E. P. Pegues, C. L. Sentman, M. T. Stephan, Biopolymer implants enhance the efficacy of adoptive T-cell therapy. *Nat. Biotechnol.* **33**, 97–101 (2015).
8. P. Sridhar, F. Petrocca, Regional delivery of chimeric antigen receptor (CAR) T-cells for cancer therapy. *Cancer* **9**, 92 (2017).
9. Q. Hu, H. Li, E. Archibong, Q. Chen, H. Ruan, S. Ahn, E. Dukhovlinova, Y. Kang, D. Wen, G. Dotti, Z. Gu, Inhibition of post-surgery tumour recurrence via a hydrogel releasing CAR-T cells and anti-PDL1-conjugated platelets. *Nat. Biomed. Eng.* **5**, 1038–1047 (2021).
10. S. Correa, A. K. Grosskopf, H. Lopez Hernandez, D. Chan, A. C. Yu, L. M. Stapleton, E. A. Appel, Translational applications of hydrogels. *Chem. Rev.* **121**, 11385–11457 (2021).
11. T. T. Smith, H. F. Moffett, S. B. Stephan, C. F. Opel, A. G. Dumigan, X. Jiang, V. G. Pillarisetty, S. P. S. Pillai, K. D. Wittrup, M. T. Stephan, Biopolymers codelivering engineered T cells

and sting agonists can eliminate heterogeneous tumors. *J. Clin. Invest.* **127**, 2176–2191 (2017).

12. T. A. Waldmann, E. Lugli, M. Roederer, L. P. Perera, J. V. Smedley, R. P. Macallister, C. K. Goldman, B. R. Bryant, J. M. Decker, T. A. Fleisher, H. C. Lane, M. C. Sneller, R. J. Kurlander, D. E. Kleiner, J. M. Pletcher, W. D. Figg, J. L. Yovandich, S. P. Creekmore, Safety (toxicity), pharmacokinetics, immunogenicity, and impact on elements of the normal immune system of recombinant human IL-15 in rhesus macaques. *Blood* **117**, 4787–4795 (2011).
13. M. T. Lotze, Y. L. Matory, A. A. Rayner, S. E. Ettinghausen, J. T. Vetto, C. A. Seipp, S. A. Rosenberg, Clinical effects and toxicity of interleukin-2 in patients with cancer. *Cancer* **58**, 2764–2772 (1986).
14. J. S. Miller, C. Morishima, D. G. McNeel, M. R. Patel, H. E. K. Kohrt, J. A. Thompson, P. M. Sondel, H. A. Wakelee, M. L. Disis, J. C. Kaiser, M. A. Cheever, H. Streicher, S. P. Creekmore, T. A. Waldmann, K. C. Conlon, A first-in-human phase I study of subcutaneous outpatient recombinant human IL15 (rhIL15) in adults with advanced solid tumors. *Clin. Cancer Res.* **24**, 1525–1535 (2018).
15. N. Momin, N. K. Mehta, N. R. Bennett, L. Ma, J. R. Palmeri, M. M. Chinn, E. A. Lutz, B. Kang, D. J. Irvine, S. Spranger, K. D. Wittrup, Anchoring of intratumorally administered cytokines to collagen safely potentiates systemic cancer immunotherapy. *Sci. Transl. Med.* **11**, eaaw2614 (2019).
16. A. A. Foster, L. M. Marquardt, S. C. Heilshorn, The diverse roles of hydrogel mechanics in injectable stem cell transplantation. *Curr. Opin. Chem. Eng.* **15**, 15–23 (2017).
17. A. K. Grosskopf, G. A. Roth, A. A. A. Smith, E. C. Gale, H. L. Hernandez, E. A. Appel, Injectable supramolecular polymer–nanoparticle hydrogels enhance human mesenchymal stem cell delivery. *Bioeng. Transl. Med.* **5**, e10147 (2020).
18. E. A. Appel, M. W. Tibbitt, M. J. Webber, B. A. Mattix, O. Veiseh, R. Langer, Self-assembled hydrogels utilizing polymer–nanoparticle interactions. *Nat. Commun.* **6**, 1–9 (2015).
19. L. M. Stapleton, A. N. Steele, H. Wang, H. Lopez Hernandez, A. C. Yu, M. J. Paulsen, A. A. A. Smith, G. A. Roth, A. D. Thakore, H. J. Lucian, K. P. Totherow, S. W. Baker, Y. Tada, J. M. Farry, A. Eskandari, C. E. Hironaka, K. J. Jaatinen, K. M. Williams, H. Bergamasco, C. Marschel, B. Chadwick, F. Grady, M. Ma, E. A. Appel, Y. J. Woo, Use of a supramolecular polymeric hydrogel as an effective post-operative pericardial adhesion barrier. *Nat. Biomed. Eng.* **3**, 611–620 (2019).
20. C. M. Meis, E. E. Salzman, C. L. Maikawa, A. A. A. Smith, J. L. Mann, A. K. Grosskopf, E. A. Appel, Self-assembled, dilution-responsive hydrogels for enhanced thermal stability of insulin biopharmaceuticals. *ACS Biomater Sci. Eng.* **7**, 4221–4229 (2021).
21. A. K. Grosskopf, O. A. Saouaf, H. Lopez Hernandez, E. A. Appel, Gelation and yielding behavior of polymer–nanoparticlehydrogels. *J. Polym. Sci. A* **59**, 2854–2866 (2021).
22. C. M. Meis, A. K. Grosskopf, S. Correa, E. A. Appel, Injectable supramolecular polymer-nanoparticle hydrogels for cell and drug delivery applications. *J. Vis. Exp.* **168**, e62234 (2021).
23. A. C. Yu, H. Chen, D. Chan, G. Agmon, L. M. Stapleton, A. M. Sevit, M. W. Tibbitt, J. D. Acosta, T. Zhang, P. W. Franzia, R. Langer, E. A. Appel, Scalable manufacturing of biomimetic moldable hydrogels for industrial applications. *Proc. Natl. Acad. Sci. U.S.A.* **113**, 14255–14260 (2016).
24. G. A. Roth, E. C. Gale, M. Alcántara-Hernández, W. Luo, E. Axpe, R. Verma, Q. Yin, A. C. Yu, H. L. Hernandez, C. L. Maikawa, A. A. A. Smith, M. M. Davis, B. Pulendran, J. Idoyaga, E. A. Appel, Injectable hydrogels for sustained codelivery of subunit vaccines enhance humoral immunity. *ACS Cent. Sci.* **6**, 1800–1812 (2020).
25. C. Y. Anthony, H. Lian, X. Kong, H. L. Hernandez, J. Qin, E. A. Appel, Physical networks from entropy-driven non-covalent interactions. *Nat. Commun.* **12**, 746 (2021).
26. H. Lopez Hernandez, J. W. Souza, E. A. Appel, A quantitative description for designing the extrudability of shear-thinning physical hydrogels. *Macromol. Biosci.* 2000295 (2020).
27. F. S. Majedi, M. M. Hasani-Sadrabadi, T. J. Thauland, S. Li, L.-S. Bouchard, M. J. Butte, T-cell activation is modulated by the 3D mechanical microenvironment. *Biomaterials* 120058 (2020).
28. B. A. Aguado, W. Mulyasasmita, J. Su, K. J. Lampe, S. C. Heilshorn, Improving viability of stem cells during syringe needle flow through the design of hydrogel cell carriers. *Tissue Eng. Part A* **18**, 806–815 (2012).
29. H. Lopez Hernandez, A. K. Grosskopf, L. M. Stapleton, G. Agmon, E. A. Appel, Non-newtonian polymer–nanoparticle hydrogels enhance cell viability during injection. *Macromol. Biosci.* **19**, 1800275 (2019).
30. D. S. Martin, M. E. Balis, B. Fisher, E. Frei, E. J. Freireich, G. H. Heppner, J. F. Holland, J. A. Houghton, P. J. Houghton, R. K. Johnson, Role of murine tumor models in cancer treatment research. *Cancer Res.* **46**, 2189–2192 (1986).
31. A. M. Ring, J. X. Lin, D. Feng, S. Mitra, M. Rickert, G. R. Bowman, V. S. Pande, P. Li, I. Moraga, R. Spolski, E. Özkan, W. J. Leonard, K. C. Garcia, Mechanistic and structural insight into the functional dichotomy between IL-2 and IL-15. *Nat. Immunol.* **13**, 1187–1195 (2012).
32. M. Varon, T. Levy, G. Mazor, H. Ben David, R. Marciano, Y. Krelin, M. Prasad, M. Elkabets, D. Pauck, U. Ahmadov, D. Picard, N. Qin, A. Borkhardt, G. Reifenberger, G. Leprivier, M. Remke, B. Rotblat, The long noncoding RNA TP73-AS1 promotes tumorigenicity of medulloblastoma cells. *Int. J. Cancer* **145**, 3402–3413 (2019).
33. J. Bunt, T. G. de Haas, N. E. Hasselt, D. A. Zwijnenburg, J. Koster, R. Versteeg, M. Kool, Regulation of cell cycle genes and induction of senescence by overexpression of OTX2 in medulloblastoma cell lines. *Mol. Cancer Res.* **8**, 1344–1357 (2010).
34. C. Graham, A. Jozwik, A. Pepper, R. Benjamin, Allogeneic CAR-T cells: More than ease of access? *Cell* **7**, 155 (2018).
35. A. B. Nair, S. Jacob, A simple practice guide for dose conversion between animals and human. *J. Basic Clin. Pharm.* **7**, 27–31 (2016).
36. K. C. Conlon, E. Lugli, H. C. Welles, S. A. Rosenberg, A. T. Fojo, J. C. Morris, T. A. Fleisher, S. P. Dubois, L. P. Perera, D. M. Stewart, C. K. Goldman, B. R. Bryant, J. M. Decker, J. Chen, T. A. Worthy, W. D. Figg Sr., C. J. Peer, M. C. Sneller, H. C. Lane, J. L. Yovandich, S. P. Creekmore, M. Roederer, T. A. Waldmann, Redistribution, hyperproliferation, activation of natural killer cells and CD8 T cells, and cytokine production during first-in-human clinical trial of recombinant human interleukin-15 in patients with cancer. *J. Clin. Oncol.* **33**, 74–82 (2015).
37. Y. Hori, A. M. Winans, C. C. Huang, E. M. Horrigan, D. J. Irvine, Injectable dendritic cell-carrying alginate gels for immunization and immunotherapy. *Biomaterials* **29**, 3671–3682 (2008).
38. D. Alizadeh, R. A. Wong, X. Yang, D. Wang, J. R. Pecoraro, C. F. Kuo, B. Aguilar, Y. Qi, D. K. Ann, R. Starr, R. Urak, X. Wang, S. J. Forman, C. E. Brown, IL15 enhances CAR-T cell antitumor activity by reducing mtorc1 activity and preserving their stem cell memory phenotype. *Cancer Immunol. Res.* **7**, 759–772 (2019).
39. L. V. Hurton, H. Singh, A. M. Najjar, K. C. Switzer, T. Mi, S. Maiti, S. Olivares, B. Rabinovich, H. Huls, M. A. Forget, V. Datar, P. Kebriaei, D. A. Lee, R. E. Champlin, L. J. N. Cooper, Tethered IL-15 augments antitumor activity and promotes a stem-cell memory subset in tumor-specific T cells. *Proc. Natl. Acad. Sci. U.S.A.* **113**, E7788–E7797 (2016).
40. W. Zhu, Z. Zhao, B. Feng, W. Yu, J. Li, H. Guo, R. Yang, CD8+ CD39+ t cells mediate anti-tumor cytotoxicity in bladder cancer. *Onco Targets Ther.* **14**, 2149–2161 (2021).
41. K. E. Kortekaas, S. J. Santegoets, G. Sturm, I. Ehsan, S. L. van Egmond, F. Finotello, Z. Trajanoski, M. J. P. Welters, M. I. E. van Poelgeest, S. H. van der Burg, CD39 identifies the $CD4^+$ tumor-specific T-cell population in human cancer. *Cancer Immunol. Res.* **8**, 1311–1321 (2020).
42. T. Duhen, R. Duhen, R. Montler, J. Moses, T. Moudgil, N. F. de Miranda, C. P. Goodall, T. C. Blair, B. A. Fox, J. E. McDermott, S. C. Chang, G. Grunkemeier, R. Leidner, R. B. Bell, A. D. Weinberg, Co-expression of CD39 and CD103 identifies tumor-reactive CD8 T cells in human solid tumors. *Nat. Commun.* **9**, 2724 (2018).
43. E. Tak, D. H. Jung, S. H. Kim, G. C. Park, D. Y. Jun, J. Lee, B. H. Jung, V. A. Kirchner, S. Hwang, G. W. Song, S. G. Lee, Protective role of hypoxia-inducible factor-1α-dependent CD39 and CD73 in fulminant acute liver failure. *Toxicol. Appl. Pharmacol.* **314**, 72–81 (2017).
44. H. K. Eltzschig, D. Köhler, T. Eckle, T. Kong, S. C. Robson, S. P. Colgan, Central role of Sp1-regulated CD39 in hypoxia/ischemia protection. *Blood* **113**, 224–232 (2009).
45. A. Coulibaly, S. Y. Velásquez, N. Kassner, J. Schulte, M. V. Barbarossa, H. A. Lindner, STAT3 governs the HIF-1α response in IL-15 primed human NK cells. *Sci. Rep.* **11**, 7023 (2021).
46. R. G. Majzner, J. L. Theruvath, A. Nellan, S. Heitzeneder, Y. Cui, C. W. Mount, S. P. Rietberg, M. H. Linde, P. Xu, C. Rota, E. Sotillo, L. Labanieh, D. W. Lee, R. J. Orentas, D. S. Dimitrov, Z. Zhu, B. S. Croix, A. Delaidelli, A. Sekunova, E. Bonvini, S. S. Mitra, M. M. Quezado, R. Majeti, M. Monje, P. H. B. Sorensen, J. M. Maris, C. L. Mackall, CAR T cells targeting B7-H3, a pan-cancer antigen, demonstrate potent preclinical activity against pediatric solid tumors and brain tumors. *Clin. Cancer Res.* **25**, 2560–2574 (2019).
47. M. P. Hall, J. Unch, B. F. Binkowski, M. P. Valley, B. L. Butler, M. G. Wood, P. Otto, K. Zimmerman, G. Vidugiris, T. Machleidt, M. B. Robers, H. A. Benink, C. T. Eggers, M. R. Slater, P. L. Meisenheimer, D. H. Klaubert, F. Fan, L. P. Encell, K. V. Wood, Engineered luciferase reporter from a deep sea shrimp utilizing a novel imidazopyrazinone substrate. *ACS Chem. Biol.* **7**, 1848–1857 (2012).
48. M. S. Hughes, Y. Y. L. Yu, M. E. Dudley, Z. Zheng, P. F. Robbins, Y. Li, J. Wunderlich, R. G. Hawley, M. Moayeri, S. A. Rosenberg, R. A. Morgan, Transfer of a tcr gene derived from a patient with a marked antitumor response conveys highly active T-cell effector functions. *Hum. Gene Ther.* **16**, 457–472 (2005).
49. J. Chu, Y. Oh, A. Sens, N. Ataie, H. Dana, J. J. Macklin, T. Laviv, E. S. Welf, K. M. Dean, F. Zhang, B. B. Kim, C. T. Tang, M. Hu, M. A. Baird, M. W. Davidson, M. A. Kay, R. Fiolka, R. Yasuda, D. S. Kim, H. L. Ng, M. Z. Lin, A bright cyan-excitable orange fluorescent protein facilitates dual-emission microscopy and enhances bioluminescence imaging in vivo. *Nat. Biotechnol.* **34**, 760–767 (2016).
50. N. C. Shaner, G. G. Lambert, A. Chammas, Y. Ni, P. J. Cranfill, M. A. Baird, B. R. Sell, J. R. Allen, R. N. Day, M. Israelsson, M. W. Davidson, J. Wang, A bright monomeric green fluorescent protein derived from branchiostoma lanceolatum. *Nat. Methods* **10**, 407–409 (2013).
51. A. J. Walker, R. G. Majzner, L. Zhang, K. Wanhainen, A. H. Long, S. M. Nguyen, P. Lopomo, M. Vigny, T. J. Fry, R. J. Orentas, C. L. Mackall, Tumor antigen and receptor densities

regulate efficacy of a chimeric antigen receptor targeting anaplastic lymphoma kinase. *Mol. Ther.* **25**, 2189–2201 (2017).
52. D. Axelrod, D. E. Koppel, J. Schlessinger, E. Elson, W. W. Webb, Mobility measurement by analysis of fluorescence photobleaching recovery kinetics. *Biophys. J.* **16**, 1055–1069 (1976).

Acknowledgments: We would like to acknowledge the Stanford Center for Innovations in In vivo Imaging (small animal imaging center), particularly J. Thanh Lee and L. J. Pisani. We would also like to acknowledge the Stanford Animal Histology Core, particularly E. Petersen. **Funding:** This work was supported by Center for Human Systems Immunology with Bill and Melinda Gates Foundation (OPP1113682), American Cancer Society (RSG-18-133-01), NSF Graduate Research Fellowships (A.K.G. and L.L.), Stanford Graduate Fellowship in Science and Engineering (A.K.G. and L.L.), Siebel Scholarship (L.L.), National Cancer Institute of the NIH (F32CA247352) (S.C.), NSERC Postgraduate Scholarship (C.L.M.), Stanford BioX Bowes Graduate Student Fellowship (C.L.M.), NIH Cell and Molecular Biology Training Program (T32 GM007276) (E.C.G.), Eastman Kodak Fellowship (B.S.O.), Schmidt Science Fellows program, in partnership with the Rhodes Trust (A.I.d.), NIH F31 (1F31CA250405-01) (O.A.), and NIH National Cancer Institute Grant (R37 CA214136) (O.C.). **Author contributions:** Conceptualization: A.K.G., E.A.A., and L.L. Methodology: A.K.G., L.L., and E.A.A. Investigation: A.K.G., L.L., G.A.R., S.C., B.S.O., O.A., C.L.Mai., A.I.d., E.C.G., J.Y., J.H.K., D.D.K., P.X., and C.K.J. Writing: A.K.G., E.A.A., L.L., O.C., C.L.Mac., and J.R.C. **Competing interests:** E.A.A., A.K.G., C.L.Mac., and L.L. are listed on a pending patent related to this work filed by Stanford University (no. PCT/US2021/055897, filed 20 October 2021). E.A.A., G.A.R., S.C., and E.C.G. are listed on a pending patent related to this work filed by Stanford University (no. PCT/US2019/054070, filed 1 October 2019). C.L.Mac. is listed on a patent related to this work filed by the United States, as represented by the Secretary of the Department of Health and Human Services (no. PCT/US2016/050887, filed 9 September 2016, published 16 March 2017). L.L. is a consultant for Lyell Immunopharma and Syncopation Life Sciences. J.R.C. is a cofounder and equity holder of Trapeze Therapeutics Inc., Combangio Inc., and Virsti Therapeutics Inc.; has financial interests in Aravive Inc., Xyence Therapeutics Inc., and Syncopation Life Sciences; and is a member of the Board of Directors of Ligand Pharmaceuticals and Revel Pharmaceuticals. C.L.Mac. is a cofounder and consultant for Immatics, NeoImmuneTech, Apricity, Nektar, Ensoma, Mammoth, GSK, Lyell, and Syncopation, which are developing CAR-based therapies. E.A.A. is a cofounder and equity holder of Surf Bio Inc. The authors declare that they have no other competing interests. **Data and materials availability:** All data needed to evaluate the conclusions in the paper are present in the paper and/or the Supplementary Materials.

Submitted 22 December 2021
Accepted 19 February 2022
Published 8 April 2022
10.1126/sciadv.abn8264
