# Supplementary material for: Delivery of CAR-T cells in a transient injectable stimulatory hydrogel niche improves treatment of solid tumors
Source: Sci Adv. 2022 Apr 8;8(14):eabn8264. doi: 10.1126/sciadv.abn8264 (PMC8993118; doi:10.1126/sciadv.abn8264)
Supplement: Supplementary file 2 — Figs. S1 to S16 Tables S1 to S3 Supplementary Discussion [file sciadv.abn8264_sm.pdf]

Science Advances
AAAS

Supplementary Materials for

Delivery of CAR-T cells in a transient injectable stimulatory hydrogel niche improves treatment of solid tumors

Abigail K. Grosskopf, Louai Labanieh, Dorota D. Klysz, Gillie A. Roth, Peng Xu, Omokolade Adebowale, Emily C. Gale, Carolyn K. Jons, John H. Klich, Jerry Yan, Caitlin L. Maikawa, Santiago Correa, Ben S. Ou, Andrea I. d'Aquino, Jennifer R. Cochran, Ovijit Chaudhuri, Crystal L. Mackall*, Eric A. Appel*

*Corresponding author. Email: eappel@stanford.edu (E.A.A.); cmackall@stanford.edu (C.LM.)

Published 8 April 2022, *Sci. Adv.* **8**, eabn8264 (2022)
DOI: 10.1126/sciadv.abn8264

This PDF file includes:

Figs. S1 to S16
Tables S1 to S3
Supplementary Discussion

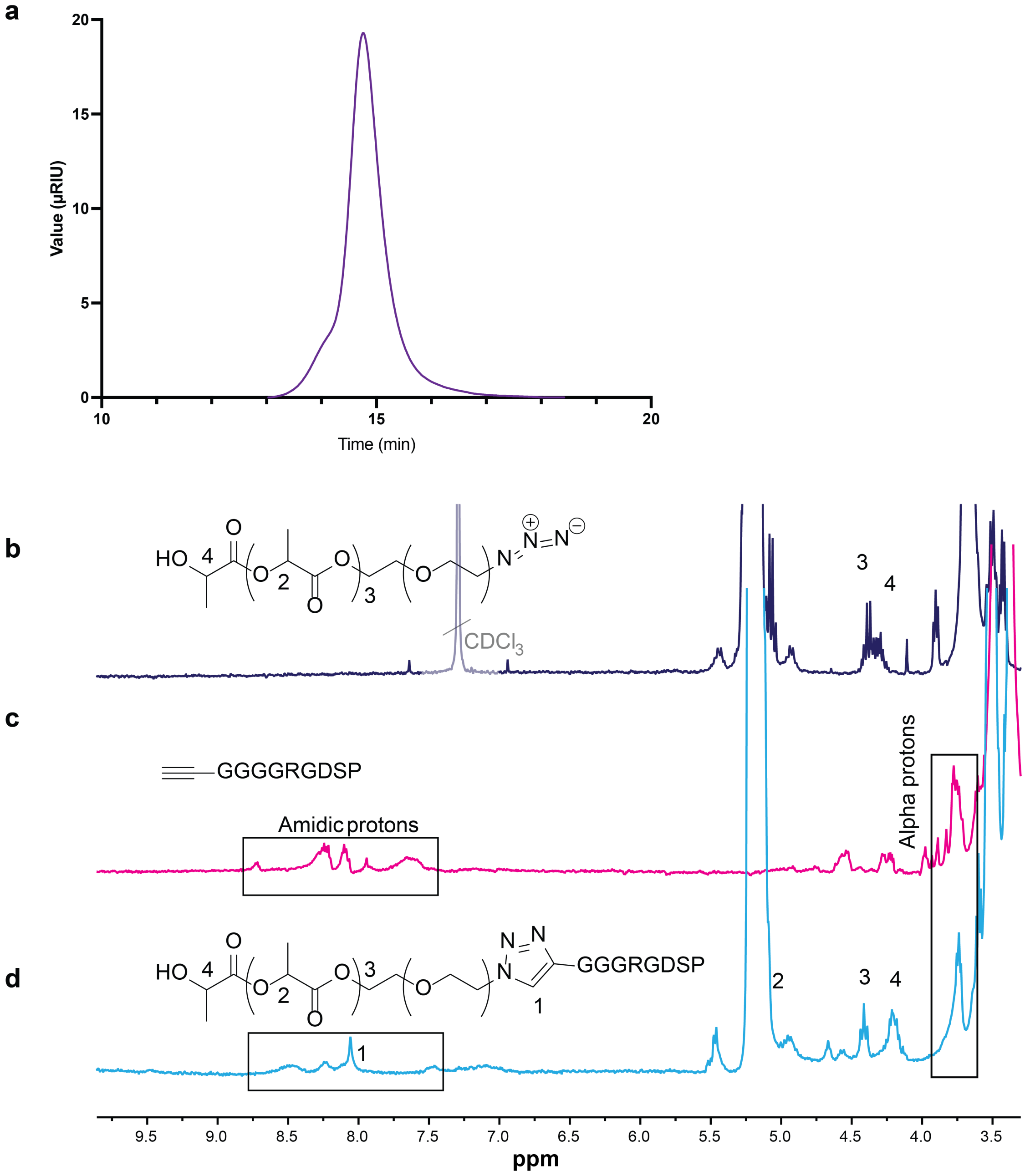

Supplementary Figure 1: Characterization of PEG-PLA. **a,** Gel permeation chromatography of the PEG-PLA polymer showing a single peak (Mn = 21 kDa; Đ = 1.08). **b,** ^{1}H-NMR ($CDCl_3$) of N_3-PEG-PLA. **c,** ^{1}H-NMR (DMSO-d_6) of propargylglycine-GGGRGDSP. **d,** ^{1}H-NMR (DMSO-d_6) of RGD-PEG-PLA copolymer showing the emergence of the triazole proton at 8.05 ppm demonstrating coupling of the RGD peptide to the PEG-PLA.

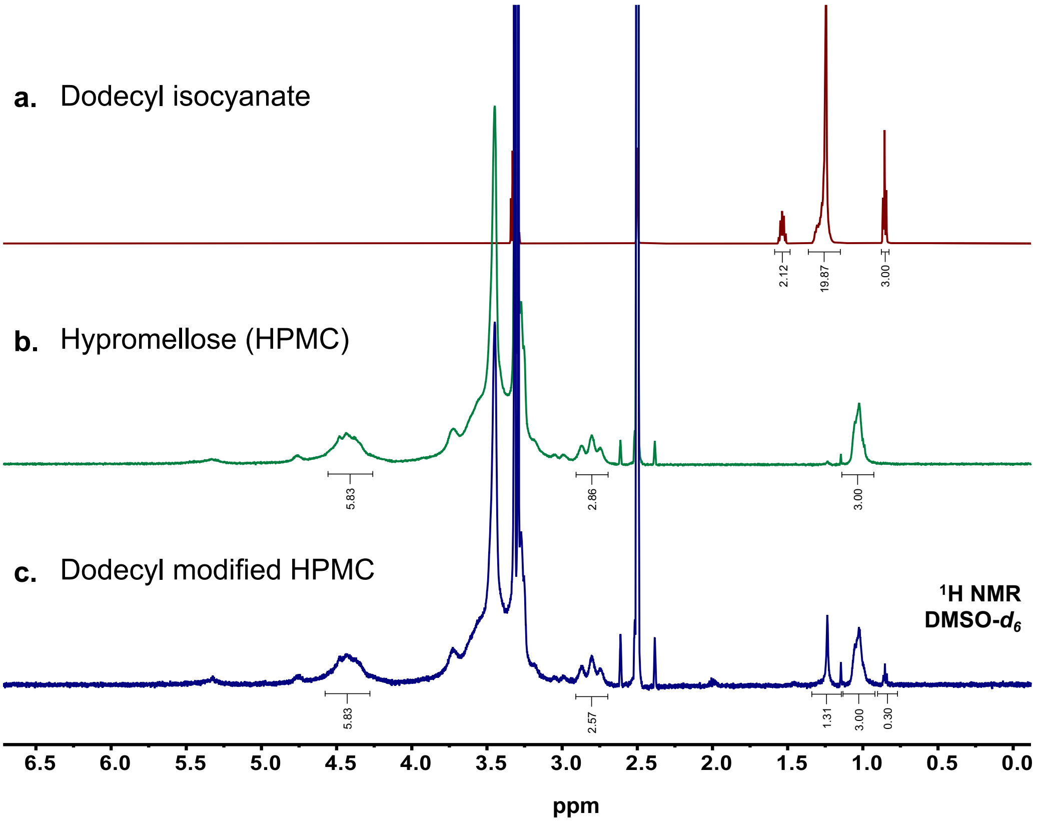

Supplementary Figure 2: Characterization of HPMC-C_{12}. **a,** ^{1}H-NMR (600 MHz, DMSO-d_6, 298 K) of dodecyliso-cyanate starting material. **b,** ^{1}H-NMR (600 MHz, DMSO-d6, 298 K) of hypromellose (HPMC) starting material. **c,** ^{1}H-NMR (600 MHz, DMSO-d_6, 298 K) of HPMC-C_{12} showing the emergence of the terminal methyl group on the dodecyl moiety at 0.86 ppm, indicating the successful coupling of the dodecyl moiety to the HPMC. Taking the ratio of the dodecyl-related peak integration at 0.86 ppm to the HPMC-related peak integration at 1 ppm, we determined a 10 mol% modification of HPMC-C_{12} (8.5 wt% modification compared to theoretical max of 10 wt% modification).

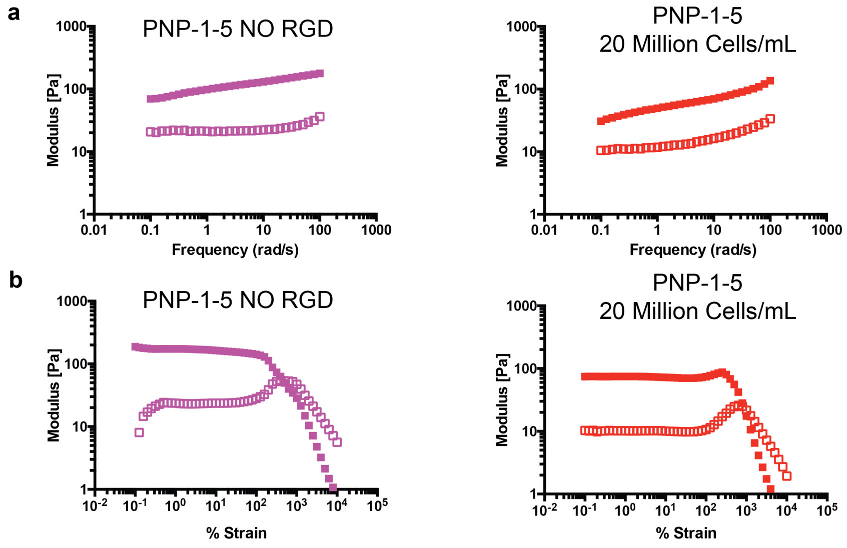

Supplementary Figure 3: Rheology of the PNP-1-5 hydrogel control formulations including controls of hydrogels without RGD-conjugated nanoparticles and hydrogels containing 20 million cells/mL. **a,** Frequency sweep (%strain=1%) for all formulations at 25°C. **b,** Amplitude sweep (ω=10 rad/s) for all formulations at 25°C.

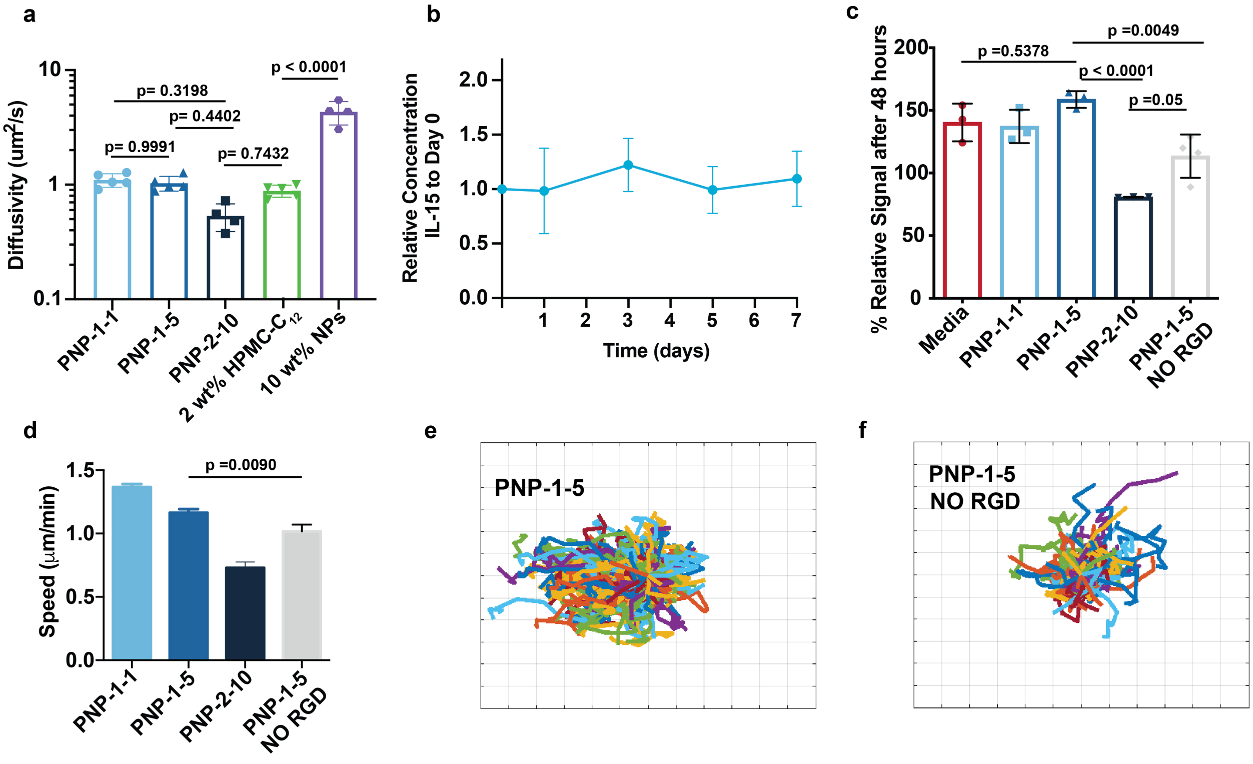

Supplementary Figure 4: *In vitro* experiments assessing diffusivity and stability IL-15 cytokine in PNP hydrogels and the benefits of RGD-conjugation for T cell proliferation and motility. **a,** Diffusivity of IL-15 in various PNP hydrogel formulations and components (first number is polymer wt%, second number is NP wt% (remaining mass is saline). **b,** Cytokine stability in PNP-1-5 hydrogel over time relative to time zero assessed by ELISA. **b,** Relative proliferation after 48 hours in PNP hydrogel formulations and 2D control. **c,** Relative proliferation of CAR-T cells in several formulations after 48 hours. **d,** CAR-T cell speeds within PNP-1-5 hydrogel formulations with and without conjugation of RGD moieties (data shown as mean±SEM). **e/f,** Trajectories of migrating CAR-T cells within indicated hydrogel formulations. The trajectories are plotted at a common origin for easy visualization. Each grid represents 50 μm.

Supplementary Table 1: Additional P values from Figure 5e survival analysis computed using a log-rank Mantel-Cox test.

Treatment pair	P value
IV, SC Saline	0.9624
IV, SC Saline + IL-15	0.2626
IV, PNP-1-5	0.023
IV, PNP-1-5 + IL-15	0.0006
SC Saline, SC Saline + IL-15	0.2954
SC Saline, PNP-1-5	0.023
SC Saline, PNP-1-5 + IL-15	0.0002
SC Saline + IL-15, PNP-1-5	0.1655

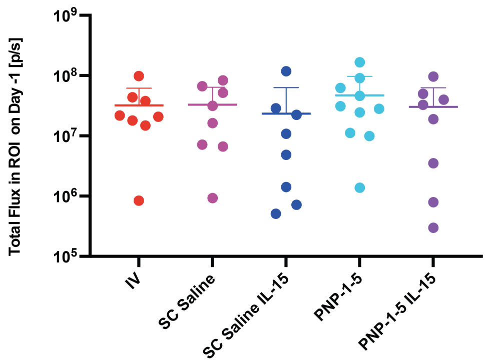

Supplementary Figure 5: Tumor burden in each group prior to administration of CAR-T cells. Mice were divided into groups the day before treatment (day -1) such that groups exhibited a similar distribution of tumor burden. No statistical significance is observed between groups.

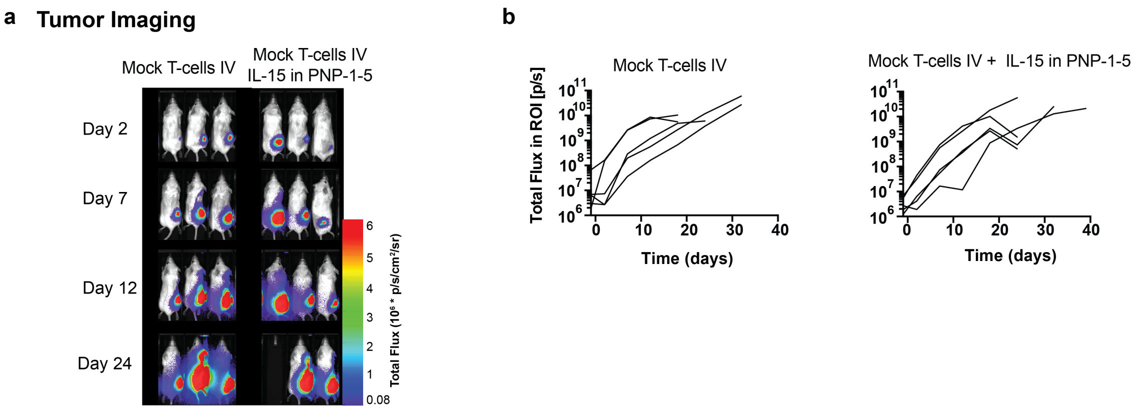

Supplementary Figure 6: Related *in vivo* controls confirming the CAR functionality is essential for treatment in the *in vivo* model. **a,** One group containing non-transduced “Mock” T cells (2 million) delivered intravenously, another group containing non-transduced “Mock” T cells (2 million) delivered intravenously with additional PNP-1-5 hydrogel with a 0.25 μg dose of IL-15 injected subcutaneously (gel not containing any cells). Tumor imaging using an *In Vivo* Imaging System. **b**, Corresponding quantification of luminescent signal from tumor imaging (n=5 for both groups from one experiment).

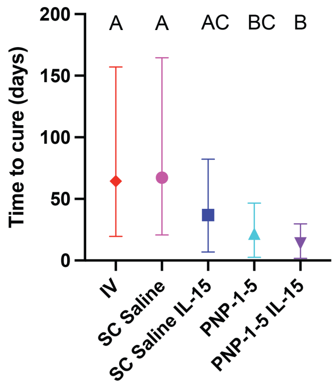

Supplementary Figure 7: Time-to-cure by treatment. CAR-T cells were administered to mice with different delivery methods, including: (i) i.v. bolus, (ii) s.c. bolus, (iii) s.c. bolus delivery containing 0.25 μg IL-15, (iv) PNP-1-5 hydrogel, and (v) PNP-1-5 hydrogel containing 0.25 μg IL-15. Time-to-cure data is shown as least-squared means and calculated using a maximum likelihood parametric regression with initial tumor size and experiment number as fixed blocking effects. The analysis indicated the time-to-cure was affected by treatment ($p < 0.0001$) and by experimental trial ($p = 0.0069$), but was not affected by initial tumor size ($p = 0.42$). Including blocking for these effects controls for these types of variation when comparing groups. Tukey-Kramer post-hoc tests were used to correct for multiple comparison. Groups not connected by the same letter are significantly different ($p<0.05$).

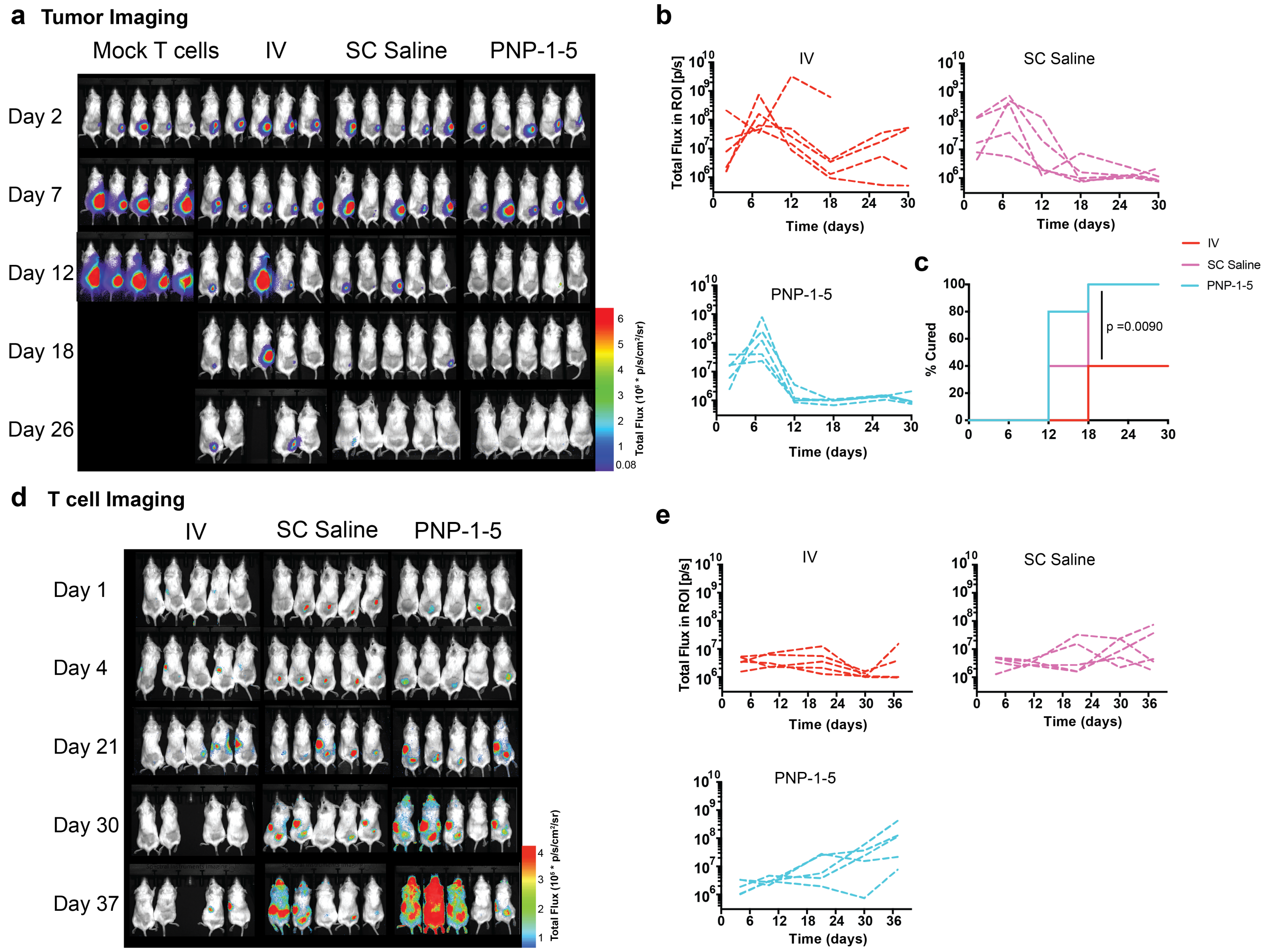

Supplementary Figure 8: *In vivo* experiment delivering 8 million CAR-T cells in PNP hydrogels and controls (cytokines not included). **a,** Tumor imaging using an *In Vivo* Imaging System (n=5 from one experiment for all groups). **b,** Corresponding quantification of luminescent signal from tumor imaging. **c,** Percentage cured during the experiment across groups (defined as time when signal drops below and stays below $2*10^6$ p/s total flux). P = 0.02 for IV compared to SC Saline. P = 0.22 for PNP-1-5 compared to SC Saline. **d,** CAR-T cell imaging using an *In Vivo* Imaging System. **e,** Corresponding quantification of luminescent signal from CAR-T cell imaging (n=5 from one experiment for all groups).

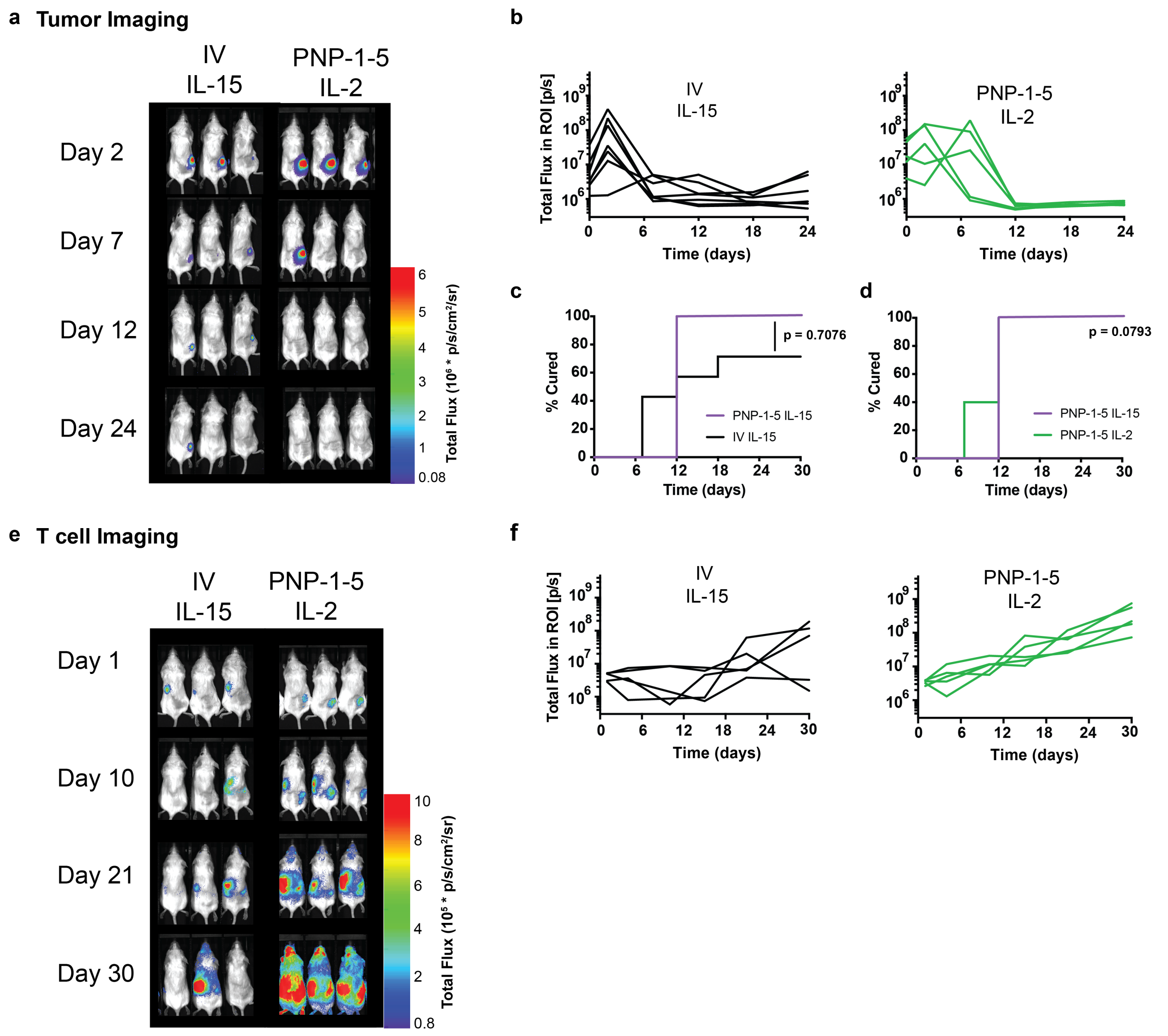

Supplementary Figure 9: Related *in vivo* controls confirming benefits to co-delivery of cells and cytokines and PNP hydrogels. This intravenous dose of IL-15 exceeds the human scaled maximum tolerated dose found in patients with cancer and would not be a translationally relevant treatment strategy (Ref 36). **a,** CAR-T cells (2 million) co-delivered with IL-15 intravenously at a 0.25 μg dose of IL-15, and CAR-T cells (2 million) encapsulated in PNP-1-5 hydrogel with a 0.25 μg dose of IL-2. Tumor imaging using an *In Vivo* Imaging System. **b,** Corresponding quantification of luminescent signal from tumor imaging (n=7 from one experiment for IV group, n=5 for PNP-1-5 with IL-2 from one experiment). **c,** Percentage cured during the experiment in the IV IL-15 group compared the PNP-1-5 IL-15 group (defined as time when signal drops below and stays below $2*10^6$ p/s total flux). **d,** Percentage cured during the experiment in the PNP-1-5 IL-2 group compared to the PNP-1-5 IL-15 group (defined as time when signal drops below and stays below $2*10^6$ p/s total flux). **e,** CAR-T cell imaging using an *In Vivo* Imaging System (n=5 for all groups). **f,** Corresponding quantification of luminescent signal from CAR-T cell imaging (n=5 for both groups from one experiment).

Supplementary Table 2: Additional P values for treatment groups from Figure 6c calculated using a one-way ANOVA with a post-hoc Tukey-Kramer multiple comparisons test.

Treatment pair	P value
IV, SC Saline	>0.9999
IV, SC Saline + IL-15	>0.9999
IV, PNP-1-5	>0.9999
SC Saline, SC Saline + IL-15	>0.9999
SC Saline, PNP-1-5	>0.9999
SC Saline + IL-15, PNP-1-5	>0.9999

Supplementary Table 3: P values for treatment groups from Figure 6g calculated using a one-way ANOVA with a post-hoc Tukey-Kramer multiple comparisons test.

Treatment pair	EM	TCM	TSCM	EF/EMRA
PNP-1-5 Mock, PNP-1-5	0.0002	0.0002	0.0038	0.0002
PNP-1-5 Mock, PNP-1-5 IL-15	<0.0001	0.1168	<0.0001	0.0028
PNP-1-5, PNP-1-5 IL-15	<0.0001	<0.0001	<0.0001	0.0269

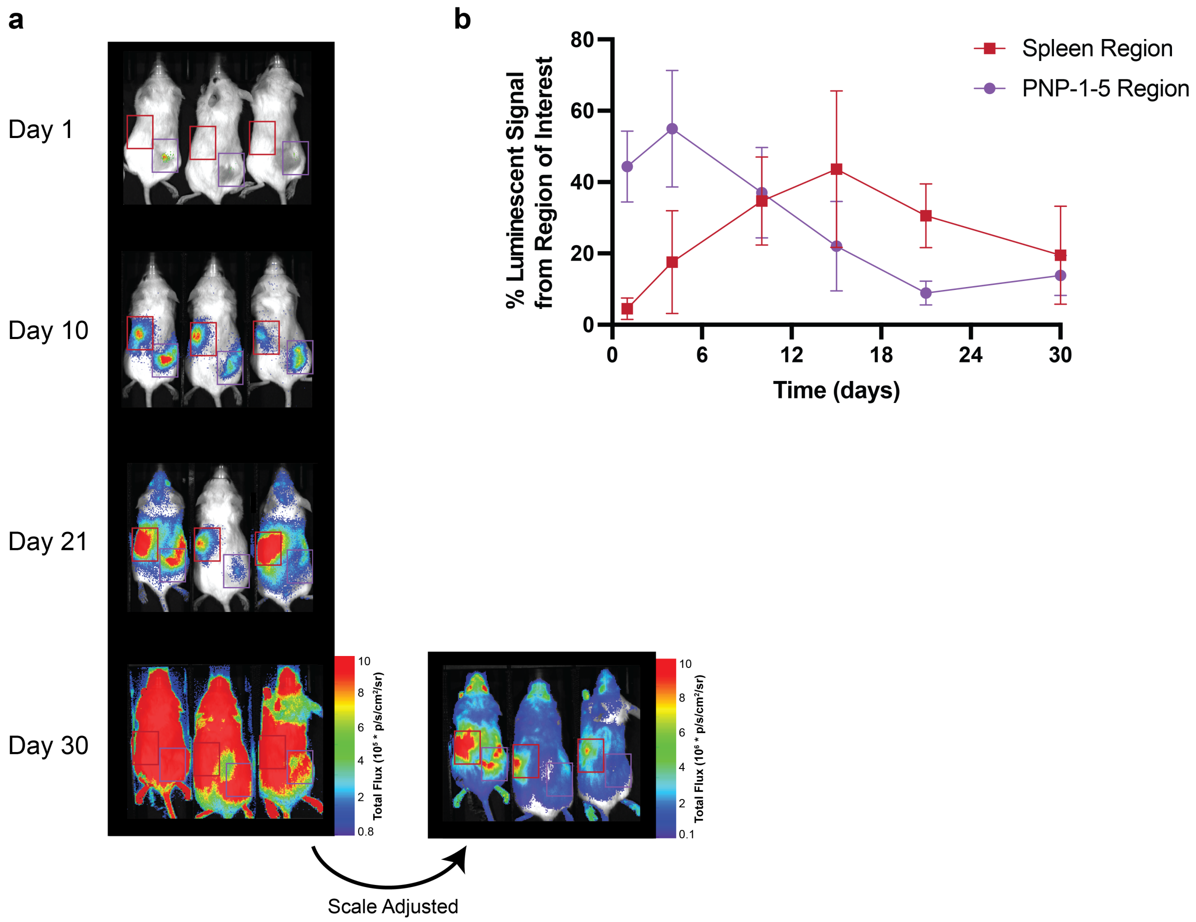

Supplementary Figure 10: Regional luminescent imaging analysis of CAR-T cells in the PNP-1-5 IL-15 group. **a,** Large-scale luminescent images of CAR-T cells of the PNP-1-5 IL-15 group. The spleen region is denoted with a red box. The region where the PNP-1-5 hydrogel was injected is denoted with a purple box. An additional Day 30 image is shown with an adjusted scale bar to clarify T-cell rich regions without saturation. **b,** Quantification of the spleen and PNP-1-5 hydrogel regions (within red and purple boxes) over time in the PNP-1-5 IL-15 group.

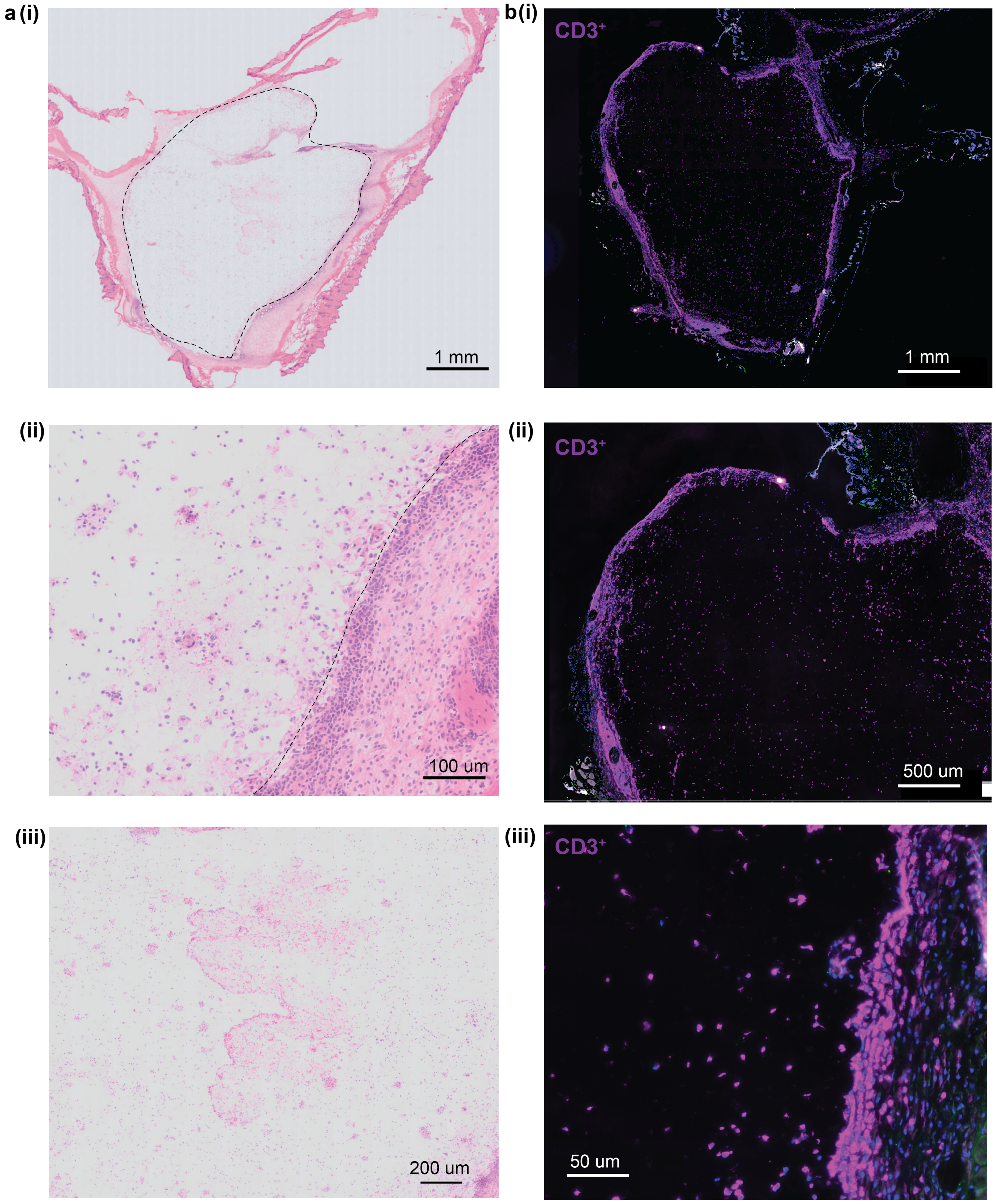

Supplementary Figure 11: Histology of explanted PNP-1-5 hydrogel containing CAR-T cells (2 million) and IL-15 after 5 days *in vivo*. **a,** Images of Hematoxylin and Eosin staining under various magnifications (indicated by scale bars). Hydrogel is outlined with black dotted lines and surrounded by exogenous tissue and cells. **b,** Images of $CD3^+$ staining in purple (Cy5, Jackson Immuno) under various magnifications (indicated by scale bars). $CD3^+$ staining includes recruited endogenous cells in addition to the delivered cells.

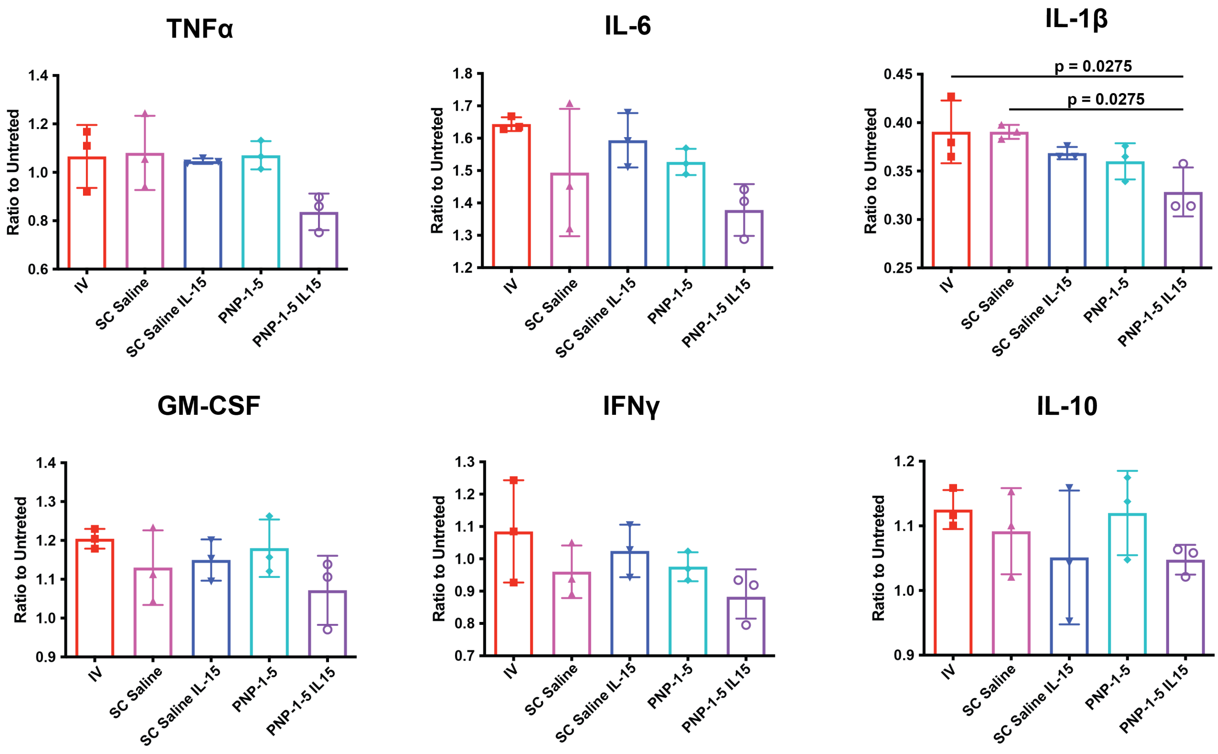

Supplementary Figure 12: Inflammatory mouse cytokines measured in the blood 3 days after treatment with CAR-T cells (2 million) in various delivery methods reported relative to values determined for naive mice (n=3 mice per group). P values are only reported here if p<0.05.

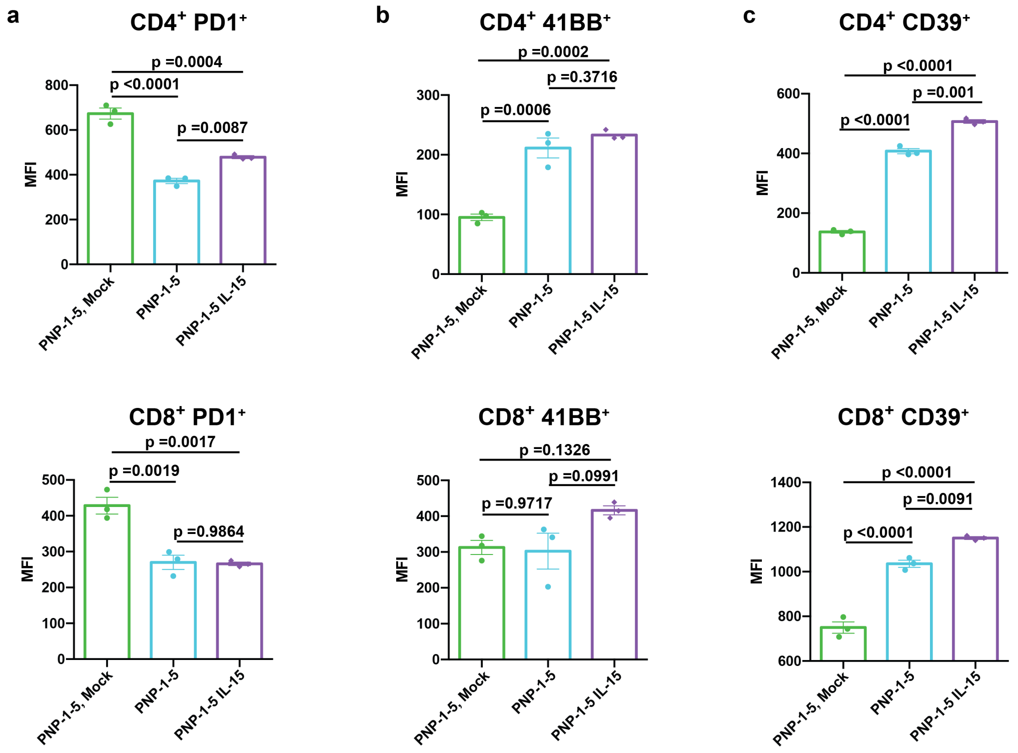

Supplementary Figure 13: Expression of T cell activation markers on CAR-T cells extracted from PNP hydrogels. MFI of **a,** PD1, **b,** 4-1BB, and **c,** CD39 staining on CAR-T cells collected 10 days after treatment in the MED8A tumor model. Data shown as mean±SEM (n=3).

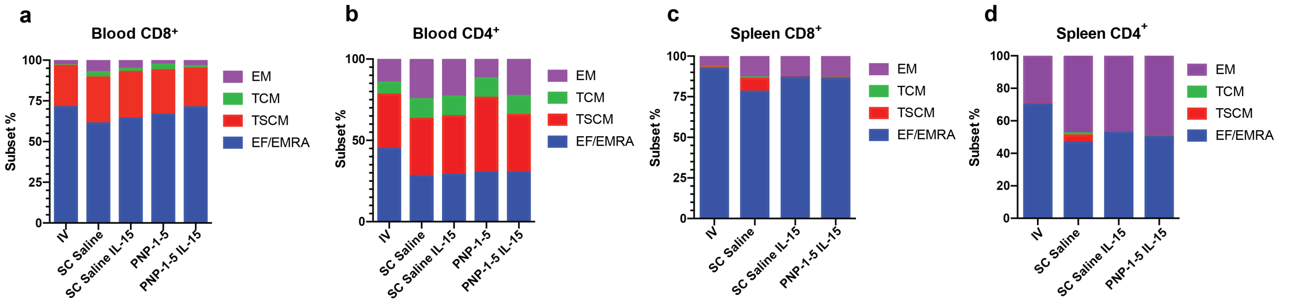

Supplementary Figure 14: T cell memory subsets from CAR-T cell treated mice. T cell memory subsets, as determined by CD62L and CD45RA staining, from **a, b,** blood and **c, d,** spleen samples collected 10 days after CAR-T cell administration in the MED8A tumor model.

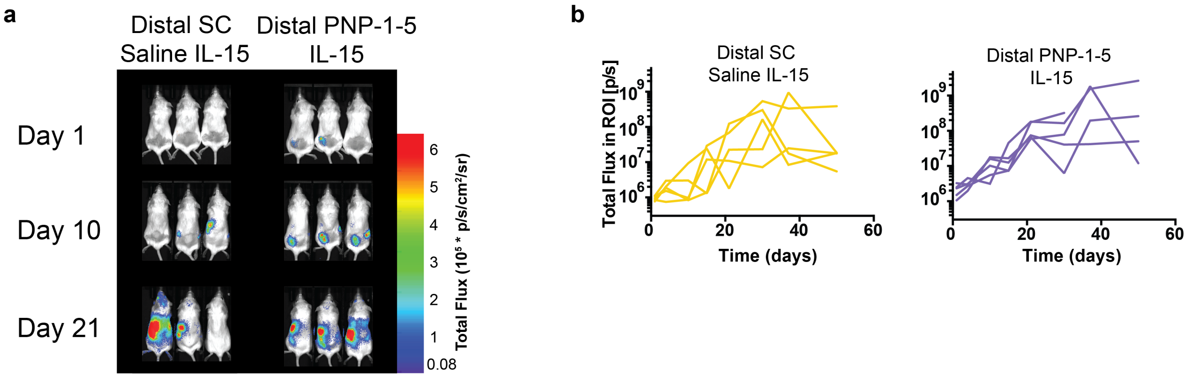

Supplementary Figure 15: *In vivo* experiment comparing CAR-T cell expansion when administered subcutaneously on the contralateral (left) flank, distal to the tumor (right subcutaneous flank). CAR-T cells (2 million) were co-administered in PNP-1-5 hydrogels or in a saline bolus at a dose of 0.25 μg/mouse IL-15. **a,** CAR-T cell imaging using an *In Vivo* Imaging System (n=5 for all groups). **b,** Corresponding quantification of luminescent signal from CAR-T cell imaging (n=5 for all groups).

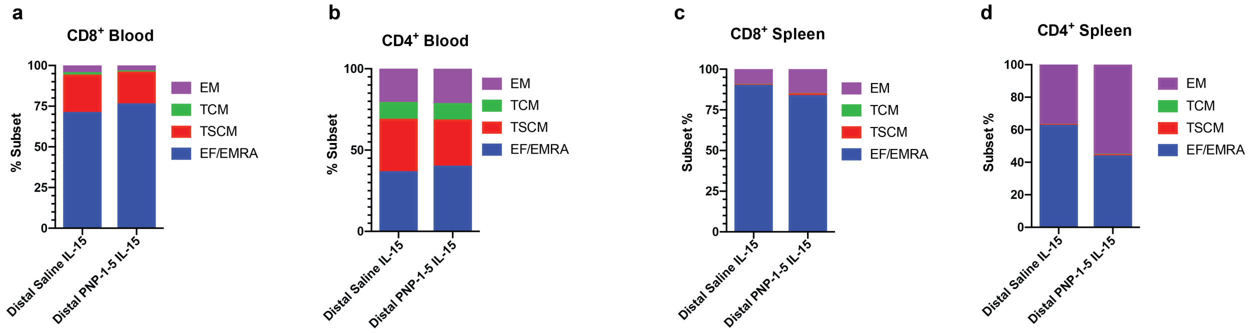

Supplementary Figure 16: CAR-T cell memory subsets from distally treated mice. CAR-T cell memory subsets, as determined by CD62L and CD45RA staining, from **a,b,** blood and **c,d,** spleen samples. CAR-T cells were collected 10 days after treatment in the MED8A tumor model. Data shown as mean±SEM (n=3).

Supplementary Discussion

Calculation of rhIL-15 Dose Equivalence in Humans

While many CAR-T therapies require lymphodepletion, there has been little research on how lymphodepletion affects the maximum tolerated dose of rhIL-15, so the dosage in our studies is based on the available literature. The maximum tolerated subcutaneous dose per day in recent human clinical trials is 2 μg/kg/day (Ref 14). We can scale this dose to mice by multiplying by 12.3, giving 24.6 μg/kg/day (Ref 35). Assuming mice are approximately 0.02 kg in weight, gives 0.492 μg/day in one dose in a mouse. We chose to administer approximately half this dose, 0.25 μg, subcutaneously in our studies. Most preclinical studies to date deliver higher doses of IL-15 (Refs 7, 9, 37). Note that even the dose we use in our studies exceeds the maximum tolerated dose found in patients with cancer of rhIL-15 intravenously delivered in humans, 0.3 μg/kg/day (Ref 36).
